# Synthesis and biological evaluation of novel cYY analogues targeting *Mycobacterium tuberculosis* CYP121A1

**DOI:** 10.1016/j.bmc.2019.02.051

**Published:** 2019-04-15

**Authors:** Safaa M. Kishk, Kirsty J. McLean, Sakshi Sood, Mohamed A. Helal, Mohamed S. Gomaa, Ismail Salama, Samia M. Mostafa, Luiz Pedro S. de Carvalho, Andrew W. Munro, Claire Simons

**Affiliations:** a School of Pharmacy & Pharmaceutical Sciences, Cardiff University, King Edward VII Avenue, Cardiff CF10 3NB, UK; b Medicinal Chemistry Department, Faculty of Pharmacy, Suez Canal University, Ismailia, Egypt; c Manchester Institute of Biotechnology, School of Chemistry, The University of Manchester, 131 Princess Street, Manchester M1 7DN, UK; d Mycobacterial Metabolism and Antibiotic Research Laboratory, The Francis Crick Institute, 1 Midland Road, London NW1 1AT, UK; e Biomedical Sciences Program, University of Science and Technology, Zewail City of Science and Technology, Giza 12588, Egypt; f Department of Chemistry, College of Clinical Pharmacy, Imam Abdulrahman Bin Faisal University, Dammam, Saudi Arabia

**Keywords:** CYP121A1, *Mycobacterium tuberculosis*, 1,4-Dibenzyl-2-imidazol-1-yl-methylpiperazine derivatives, Binding affinity assays, Molecular modelling

## Abstract

The rise in multidrug resistant (MDR) cases of tuberculosis (TB) has led to the need for the development of TB drugs with different mechanisms of action. The genome sequence of *Mycobacterium tuberculosis* (*Mtb*) revealed twenty different genes coding for cytochrome P450s. CYP121A1 catalyzes a C—C crosslinking reaction of dicyclotyrosine (cYY) producing mycocyclosin and current research suggests that either mycocyclosin is essential or the overproduction of cYY is toxic to *Mtb*. A series of 1,4-dibenzyl-2-imidazol-1-yl-methylpiperazine derivatives were designed and synthesised as cYY mimics. The derivatives substituted in the 4-position of the phenyl rings with halides or alkyl group showed promising antimycobacterial activity (MIC 6.25 μg/mL), with the more lipophilic branched alkyl derivatives displaying optimal binding affinity with CYP121A1 (^i^Pr *K*_D_ = 1.6 μM; ^t^Bu *K*_D_ = 1.2 μM). Computational studies revealed two possible binding modes within the CYP121A1 active site both of which would effectively block cYY from binding.

## Graphical abstract

**Figure:**
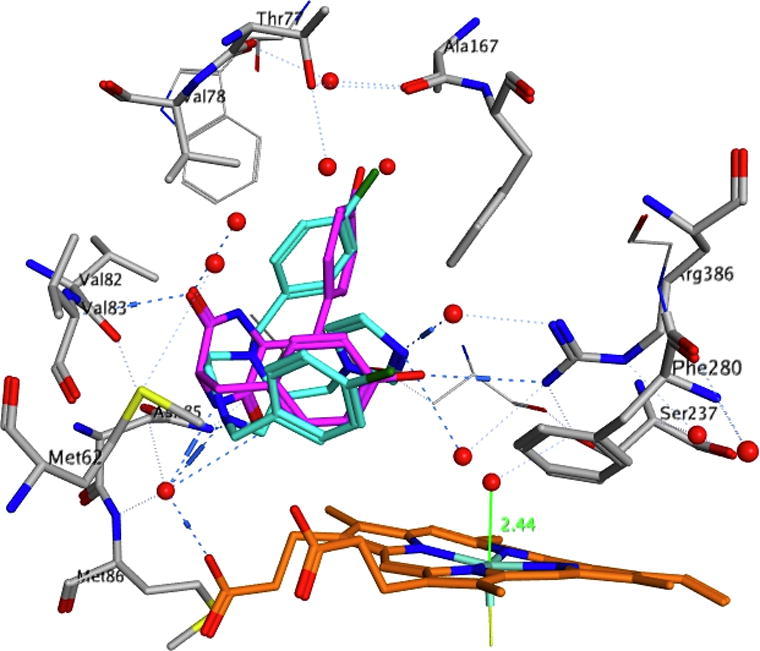

## 1 Introduction

Tuberculosis (TB) is the ninth leading cause of death worldwide, ranking above Human Immunodeficiency Virus (HIV/AIDS). In 2017, the estimated TB deaths were 1.3 million among HIV-negative people and 300,000 among HIV-positive people, and the estimated incident TB cases were 10.0 million worldwide.^1^ The standard therapeutic regimens involve the combination of four first-line drugs, often isoniazid, rifampicin, pyrazinamide and either streptomycin or ethambutol, depending upon whether there is a latent or active type of infection.^2^ Although the current medications for treating drug-sensitive TB are effective when there is optimum adherence of patients, a common problem in real-life conditions appears with TB-infected patients who generally do not adhere strictly to dosages.^3^, ^4^

The duration for treating drug-sensitive TB is usually 6 months with the four most effective agents from the first-line oral drugs administered in a single prescription for the first two months of treatment, and two of the four taken for a subsequent four months in the continuation phase, resulting in patient adherence issues.^5^ When administered in sub-optimum conditions, chronic cases of infectious drug-resistant TB appear. Long treatment durations are usually required because *Mycobacterium tuberculosis* (*Mtb*), the causative pathogen, can develop a dormancy phenotype under nutrient depletion and anaerobiosis conditions which is tolerant to several anti-TB drugs.^6^, ^7^ The development of novel anti-TB drugs has become a priority in view of the increasing global incidence of strains resistant to at least rifampicin and isoniazid (MDR-TB), or to rifampicin, isoniazid and one of the injectable second-line anti-TB drugs as well as to any of the fluoroquinolone drug series (XDR-TB),^1^ in addition to the interactions between anti-TB drugs and antiretroviral medications, and the need to treat latent TB-infected patients before the bacteria transform into their active form.^8^

Studies following the unraveling of the genome sequence of the virulent *Mtb* H37Rv strain revealed numerous genes with unknown functions.^9^ Among these were twenty different cytochrome P450 (CYP) genes coding for P450s.^10^ This large number of CYP genes was uncommon for a bacterium and, as a result, studies over the past twenty years have focused on characterizing these *Mtb* P450s, several of which play crucial roles in the survival of *Mtb*. Subsequent genome studies with *M. smegmatis* revealed several P450s, confirming the multiple important functions for the P450s in the actinobacteria.^11^

**Fig. 1:**
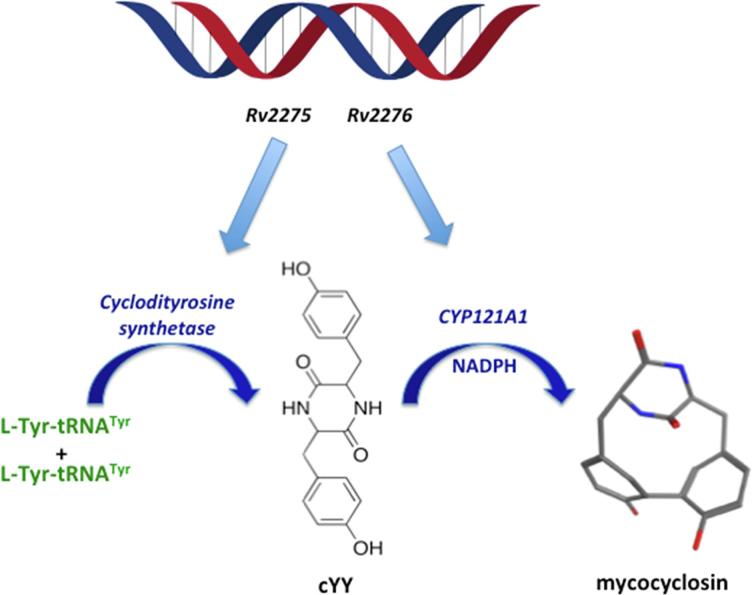
Cyclodityrosine synthase and CYP121A1 reactions to produce the cyclic dipeptide, cyclo-*L*-Tyr-*L*-Tyr (cYY), and the novel secondary metabolite mycocyclosin.

CYP121A1 (mycocyclosin synthase) was shown to be essential for bacterial growth by *in vitro* gene knockout studies.^12^, ^13^, ^14^ Also, CYP121A1, along with CYP128A1 and CYP141A1, are the only *Mtb* P450s that appear to be conserved within members of the *Mtb* complex because their homologues do not appear in the genomes of the other members of the actinobacteria family. The first evidence of CYP121A1 function in *Mtb* was derived from its gene position, which is located in an operon harboring two enzymes involved in the formation of cyclodi-l-tyrosine (cYY). The first enzyme is a cyclodipeptide synthase (encoded by *Rv2275*), which uses aminoacyl-tRNA synthetases (l-tyrosyl-tRNA^Tyr^) to catalyse the ATP-independent formation of cYY (Fig. 1).^15^, ^16^ Then, CYP121A1 (encoded by *Rv2276*) catalyzes a C—C crosslinking reaction between the respective carbons in the *ortho* position of the phenolic hydroxyl of cYY, producing mycocyclosin (Fig. 1). *Rv2276* was found to be an essential *Mtb* gene, and it was suggested that either mycocyclosin was essential or the overproduction of cYY is toxic.^14^, ^17^ Assays conducted on CYP121A1 utilising a ferredoxin and ferredoxin reductase system demonstrated that CYP121A1 catalyzes multiple turnovers of cYY in a complicated multistep process to form mycocyclosin as the single major product in the presence of NADPH.^17^

**Fig. 2:**
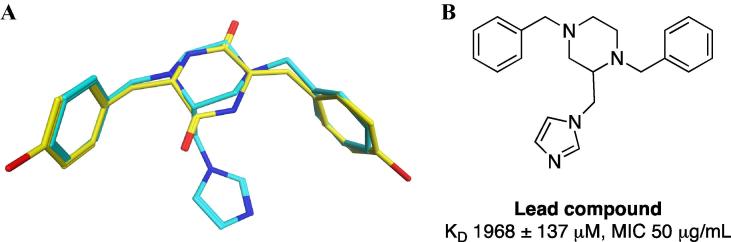
(A) Flexible alignment of cYY (yellow) and lead compound (cyan) showing overlap (B) Binding affinity and MIC data for lead compound.

In the development of CYP121A1 inhibitors, we have described several series of compounds with chemical scaffolds that mimic the natural substrate cYY.^18^, ^19^ From these series 1,4-dibenzyl-2-imidazol-1-yl-methylpiperazine derivatives were identified as potential leads for further development.^18^ Flexible alignment of these derivatives with cYY showed good overlap (Fig. 2) suggesting they would occupy a similar area as cYY within the CYP121A1 active site, while the addition of an azole haem-binding group (imidazole) may allow direct interaction between the compound and the haem iron.

The lead compound showed weak binding affinity, as determined by UV-vis optical titration, and modest inhibitory activity against *Mtb* (Fig. 2). Therefore, the aim of this study was to develop this series of compounds, through varying the substituent on the benzene rings, and to explore the structure-activity relationship to improve both binding affinity with CYP121A1 and MIC against *Mtb*.

## 2 Results and discussion

### 2.1 Chemistry

**Scheme 1:**
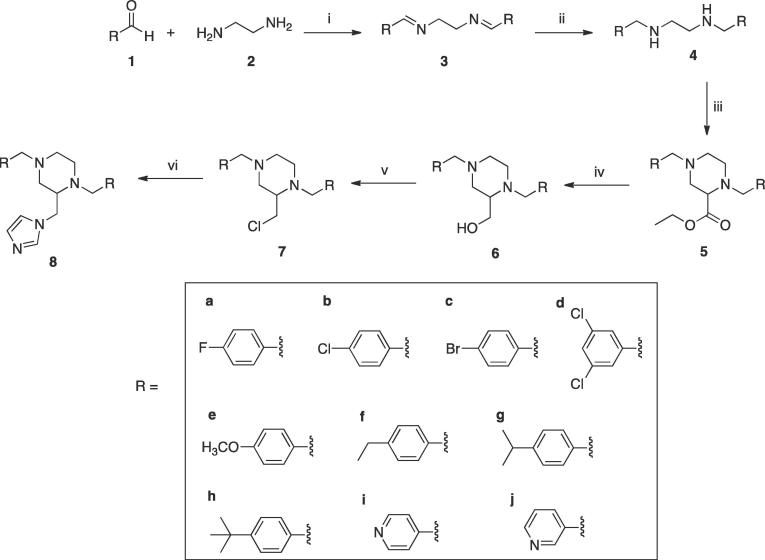
Reagents and conditions: (i) EtOH, r.t., o/n (ii) NaBH_4_, MeOH, r.t., 4 h (iii) Et_3_N, 2,3-dibromopropionic acid ethyl ester, toluene, 80 °C, o/n (iv) LiAlH_4_, THF, r.t., o/n (v) SOCl_2_, CH_2_Cl_2_, r.t., 48 h (vi) (a) Imidazole, K_2_CO_3_, CH_3_CN, 45 °C, 1 h (b) chloride **7**, 70 °C, 48 h.

**Fig. 3:**
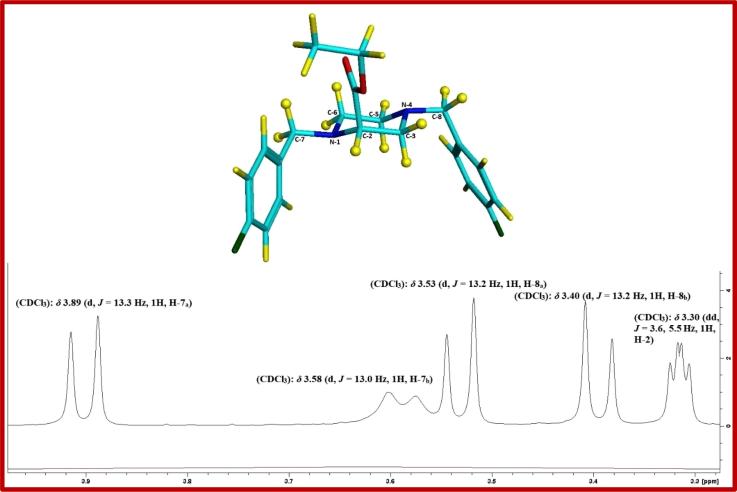
Ethyl 1,4-bis(4-fluorobenzyl)piperazine-2-carboxylate (**5a**) chair conformation with vicinal and geminal couplings of H-2, H-7 and H-8 protons.

The imidazole products (**8**) were obtained from a six-step synthetic route (Scheme 1). Substituted benzaldehyde derivatives (1) were reacted with ethylenediamine (2) in ethanol for 6 h at room temperature to give the di-Schiff base (di-imine) (**3**), which precipitated from the reaction as pure crystalline solids in high yields of 81–99%. The imine bond was reduced by NaBH_4_ followed by careful aqueous work-up while cooling in an ice bath to give the diamines (**4**) in good yields (75–97%) with the exception of the pyridinyl derivatives (**4i** and **4j**), which were obtained in yields of 29 and 31% respectively owing to their water solubility. The synthesised diamines (**4**) on reaction with ethyl 2,3-dibromopropionate^20^ resulted in the formation of ethyl 1,4-bis(substituted) piperazine-2-carboxylate (**5**) with yields in the range of 65–85%. Piperazine, as a six-membered cyclic system, can exist in both chair and boat conformations. The lowest energy and most stable conformation for the synthesised esters, using the conformational import algorithm^21^ in MOE^22^ followed by optimisation using the PM3 model Hamiltonian^23^ is shown in Fig. 3.

From the proposed 3D structure, the axial conformation for the ester moiety at C-2 was preferred,^24^ this conformation of the ester intermediates was analysed by ^1^H NMR and 2D COSY experiments. The H-2 proton had vicinal three-bond couplings across single bonds with both axial and equatorial H-3 protons, and appeared as doublet of doublets (dd) in the ^1^H NMR spectra in derivatives **5a**, **5c**, **5e**, **5h** and **5i** with coupling constants in the range of 3.4–3.6 Hz for the vicinal equatorial-equatorial couplings, and larger values in the range of 5.5–6.0 Hz for the vicinal equatorial-axial couplings. In some derivatives, the H-2 proton couplings appeared as a multiplet (m) as seen in **5b** and **5d**, or interfered with other protons as seen in **5f**, **5g** and **5j**. Geminal spin-spin couplings were conserved between the H-7 protons and the H-8 protons, and in the ^1^H NMR spectra, each proton appeared as a doublet (d) with a geminal coupling constant in the range of 13.0–14.0 Hz.

The synthesised esters (**5**) were reduced to the corresponding primary alcohols (**6**) by LiAlH_4_ after overnight stirring at room temperature with yields in the range of 56–91% obtained. Conversion of the alcohols (**6**) to the alkyl chlorides proceeded through a nucleophilic substitution S_N_2 mechanism using thionyl chloride (10 equivalents) at room temperature for 48 h. A trial to accelerate the chlorination reaction rate was done by refluxing the synthesised alcohol with thionyl chloride (2 equivalents) at 80 °C, but complex mixtures were obtained, and the products could not be separated or identified upon refluxing, especially for the halide derivatives (**7a**–**7d**). The yields of the chloride products (**7**) from reaction at room temperature for 48 h were in the range of 50–88%. The imidazolate ion, which acts as a nucleophile, was formed *in situ* by reacting imidazole with a base, for 1 h at 45 °C in acetonitrile, followed by a S_N_2 reaction between the imidazolate and the synthesised alkyl chloride (**7**). When sodium hydride (60% dispersion in mineral oil) was used as a base according to the reported method,^18^ a complex mixture was obtained, and the product could only be separated in a very low yield (<10%). Potassium carbonate was selected as the optimum base, and the reaction was complete after refluxing at 70 °C for 48 h with yields in the range of 51–69%.

**Fig. 4:**
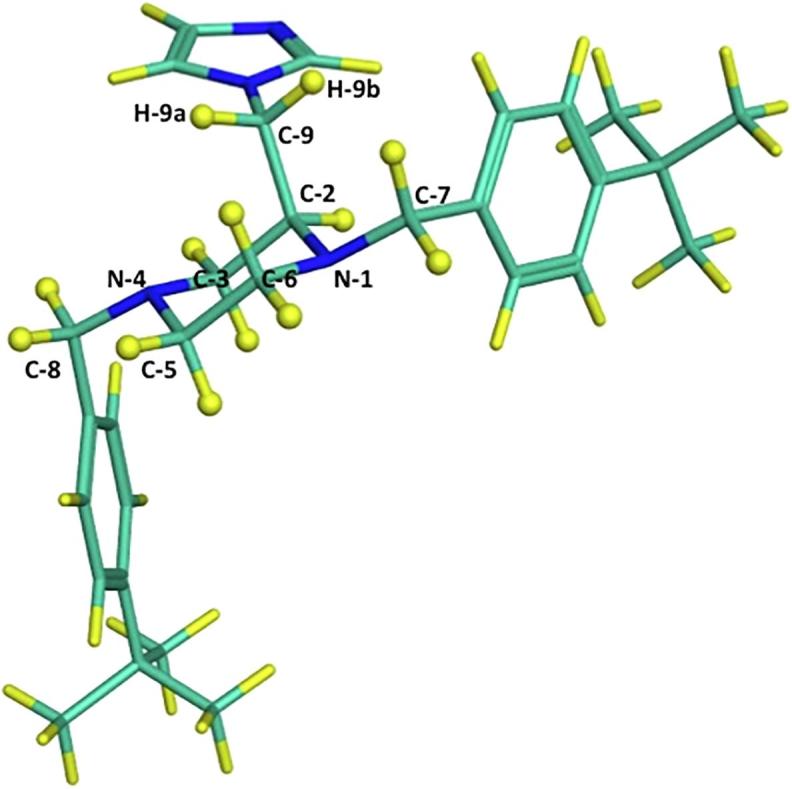
2-((1*H*-Imidazol-1-yl)methyl)-1,4-bis(4-(*tert*-butyl)benzyl)piperazine (**8h**) chair conformation after energy minimisations to explain vicinal and geminal couplings between H-9, H-2, H-7, H-8 protons.

A characteristic feature for the final imidazoles was the geminal and vicinal proton-proton coupling. The doublet of doublets (dd) at 4.28–4.29 ppm was assigned to the geminal (*J* = 13.8–14.0 Hz) and vicinal (*J* = 4.3–4.4 Hz) couplings of the H-9_a_ proton with H-9_b_ and H-2 protons, respectively. The doublet of doublets at 4.20–4.21 ppm was assigned to the geminal (*J* = 13.8–14.0 Hz) and vicinal (*J* = 7.6–8.0 Hz) coupling of the H-9_b_ proton with H-9_a_ and H-2 protons, respectively. The vicinal spin-spin coupling constants illustrated the difference in the chemical shifts between H-9_a_ and H-9_b_ protons. Vicinal spin-spin coupling constants were generally in the range of 6–8 Hz and, as the proton was closer to the imidazole group (electron-deficient group), smaller vicinal *J* values were noticed. From the proposed 3D-structure after energy minimisations (Fig. 4), the H-9_a_ proton appeared to be closer to the imidazole group than the H-9_b_ proton. Consequently, the H-9_a_ proton was less shielded and its vicinal coupling constant was less than 6.0 Hz while the H-9_b_ proton was more shielded and its vicinal coupling constant was within the range of 6–8 Hz.

### 2.2 CYP121A1 ligand binding affinity

**Fig. 5:**
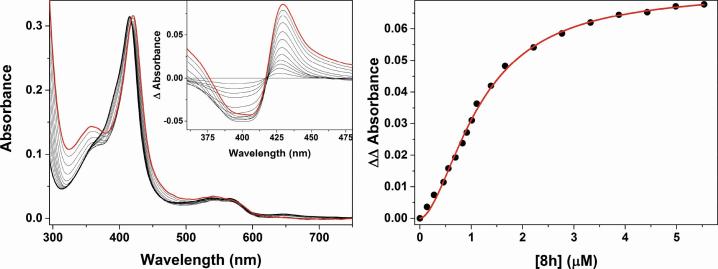
UV–Vis optical binding titration for compound **8h** binding to CYP121A1. The left hand panel shows data from a compound **8h** titration with CYP121A1 (∼4.7 μM) with the ligand-free spectrum as a thick black line, spectra following progressive additions of **8h** as thin solid lines, and the final near-saturated protein spectrum shown as a thick red line. The inset shows overlaid difference spectra generated by the subtraction of the starting spectrum from each consecutive ligand-bound spectrum collected in the titration. The right hand panel shows a plot of compound **8h**-induced absorbance change – calculated as the difference between the peak and trough in the difference spectra in the left hand panel, using the same wavelength pair (429 and 392 nm, respectively) throughout. Data were fitted using the Hill equation to give a **8h***K*_D_ value of 1.2 ± 0.1 μM.

The CYP121A1 binding affinity (*K*_D_) of the various compounds was determined by UV–vis optical titration.^25^ Binding titrations were carried out for the synthesised imidazoles (**8**), which showed a type II (inhibitor-like) red shift in the haem Soret peak to a longer wavelength (Fig. 5, for example **8h**) indicating that, in the solution state, most of these compounds coordinate either (i) directly to the CYP121A1 haem iron or (ii) indirectly to the haem iron through an interstitial water molecule.

Halogenated derivatives (**8b**–**8d**) displayed moderate binding affinity with the best *K*_D_ value observed for the 3,5-diCl compound **8d**. The 4-F compound **8a** was noted to undergo photo- and thermal-decomposition in this assay so the data obtained was not reliable.

The lipophilic derivatives had the best binding of the imidazole nitrogen to the haem iron with *K*_D_ values of 3.7 ± 0.1, 1.6 ± 0.1 and 1.2 ± 0.1 μM for **8f**, **8g** and **8h**, respectively, while the natural substrate cYY had a *K*_D_ value of 5.8 ± 0.2 μM. The moderately hydrophilic 4-OMe (**8e**) and pyridines (**8i** and **8j**) had weak binding affinity to CYP121A1 (Table 1). Compounds **8f** and **8 g** induce the most extensive Soret absorbance shifts (416.5 to 422.5/423.5 nm), suggesting that the CYP121A1 haem iron is predominantly coordinated by a direct imidazole nitrogen bond in these cases. Compounds **8b**–**8e** and **8h**–**8j** show less extensive Soret red shifts on binding the CYP121A1 haem, suggesting that ligation modes in these cases may involve imidazole nitrogen interactions with haem iron mediated through an interstitial water ligand.

**Table 1:** Binding affinity (K_D_) values for compounds against *M. tuberculosis* H37Rv.

**Figure:**
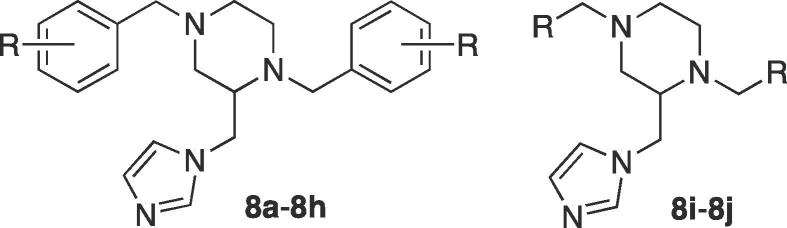

Compound	R	*K*_D_ (μM)^a^	Soret peak shift (nm)
**Lead**	H	1968 ± 137	416.5–417.5
**8b**	4-Cl	26.8 ± 7.9	416.5–419
**8c**	4-Br	11.9 ± 2.1	416.5–421
**8d**	3,5-diCl	8.1 ± 0.4	416.5–419
**8e**	4-OCH_3_	42.0 ± 14.3	416.5–419.5
**8f**	4-CH_2_CH_3_	3.7 ± 0.1	416.5–423.5
**8g**	4-CH(CH_3_)_2_	1.6 ± 0.1	416.5–422.5
**8h**	4-C(CH_3_)_3_	1.2 ± 0.1	416.5–419.5
**8i**	Pyridin-4-yl	39.2 ± 7.9	416.5–419.5
**8j**	Pyridin-3-yl	489 ± 27	416.5–421.5
**Fluconazole**		8.6 ± 0.2	
**Clotrimazole**		0.07 ± 0.01	
**cYY**		5.8 ± 0.2	

a Compound **8a** (R = 4-F) noted to undergo photo- and thermal-decomposition during this assay.

### 2.3 MIC determination against *Mycobacterium tuberculosis*

The derivatives were screened against *M. tuberculosis* H37Rv by the REMA (Resazurin Microtiter Assay) method.^26^ With the exception of the pyridyl compounds **8i** and **8j**, which were inactive (MIC > 100 μg/mL), all the compounds were more active than the lead parent compound (MIC 50 μg/mL). In the halogenated series, the 4-monosubstituted benzene ring derivatives **8a**-**8c** showed good inhibitory activity (MIC 6.25 μg/mL) compared with the 3,5-dichloro substituted derivative **8d** (MIC 25 μg/mL). The alkyl substituted series all showed promising inhibitory activity (MIC 6.25 μg/mL), while the more hydrophilic 4-methoxy derivative (**8e**) was less effective (MIC 25 μg/mL).

### 2.4 Molecular modelling

Molecular modelling of the compounds was performed using Molecular Operating Environment (MOE) sofware and the crystal structure of cYY co-crystallised with CYP121A1 (PDB 3G5H).^17^ Most of the compounds displayed two binding modes when docked within the CYP121A1 active type, both of which show indirect binding with the haem via an interstitial water molecule.

**Fig. 6:**
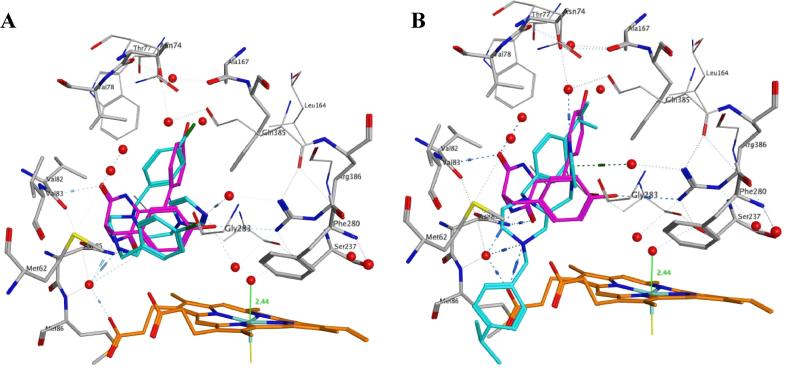
Docking of representative piperazines (cyan) (A) **8b** in binding mode 1 and (B) **8g** in binding mode 2 in the CYP121A1 active site compared with cYY (magenta). [Haem (orange), water molecules (red spheres)].

The first docking mode (mode 1) mimicked the cYY conformation as illustrated by the chloro derivative **8b** (Fig. 6A). The most consistent binding interactions were observed between the imidazole ring and Arg386 via a water molecule and the protonated piperazine NH+ and Met86 effectively bridging the active site above the haem (Table 2). In the second binding mode (mode 2) a more open/flexible conformation was observed as illustrated by the isopropyl derivative **8g** (Fig. 6B). This increased flexibility is possible owing to the piperazine ring which can exist in both chair and boat conformations. In mode 2, binding interactions were observed between the imidazole and a number of different amino acids (e.g. Asn74, Gly283, Gln385 and Arg386) via a water molecule with additional binding between the protonated piperazine NH+ and Met86. The pyridine derivatives **8i** and **8j** formed additional H-bonding interactions through the two pyridine rings. Multiple hydrophobic interactions were observed between the piperazines (**8**) for both binding modes 1 and 2 including residues Met62, Thr77, Val78, Val82, Val83, Asn85, Leu164, Ala167, Phe168, Thr229, Ala233, Gly232, Phe280 and Leu284. For the unsubstituted lead compound and the fluoro (**8a**), chloro (**8b**) and pyridine (**8i** and **8j**) derivatives, binding mode 1 was energetically prefered (Table 3).

**Table 2:** MIC values for compounds against *M. tuberculosis* H37Rv.

**Figure:**
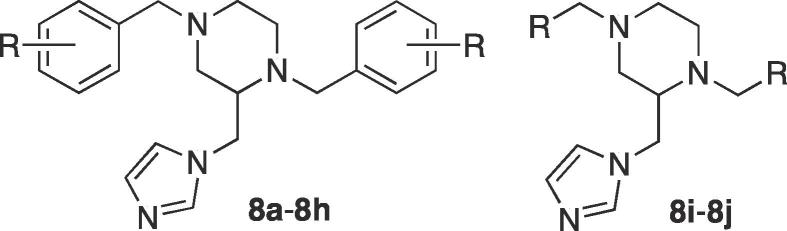

Compound	R	Mol.Wt.	MIC (μg/mL)	cLogP^a^
**Lead**	H	346.469	50	3.56
**8a**	4-F	382.450	6.25	3.87
**8b**	4-Cl	415.359	6.25	4.67
**8c**	4-Br	504.261	6.25	5.21
**8d**	3,5-diCl	484.249	25	5.79
**8e**	4-OCH_3_	406.521	25	3.3
**8f**	4-CH_2_CH_3_	402.575	6.25	5.36
**8g**	4-CH(CH_3_)_2_	430.628	6.25	6.02
**8h**	4-C(CH_3_)_3_	458.681	6.25	6.97
**8i**	Pyridin-4-yl	348.445	>100	0.88
**8j**	Pyridin-3-yl	348.445	>100	0.88
**Fluconazole**		306.271	>100	0.87
**Clotrimazole**		344.837	20	5.97
**cYY**		326.347	–	1.26

a cLogP was determined using Crippen’s fragmentation.

**Table 3:** Computational binding energies of piperazines **8** and CYP121A1.

Compound	R	Mode 1 S-value (kcal/mol)	Mode 2 S-value (kcal/mol)
**Lead**	H	−4.69	−2.97
**8a**	4-F	−6.29	−4.61
**8b**	4-Cl	−4.53	−4.23
**8c**	4-Br	–	−2.78
**8d**	3,5-diCl	–	−3.64
**8e**	4-OCH_3_	−4.73	−4.79
**8f**	4-CH_2_CH_3_	−3.34	−4.29
**8 g**	4-CH(CH_3_)_2_	−3.18	−4.21
**8 h**	4-C(CH_3_)_3_	–	0.04
**8i**	Pyridin-4-yl	−5.00	−2.35
**8j**	Pyridin-3-yl	−5.32/-6.58	−4.26

S-value = lowest binding energy.

The methoxy derivative (**8e**) showed comparable S-values for binding modes 1 and 2, while the ethyl (**8f**) and isopropyl (**8g**) derivatives had improved S-values for binding mode 2 (Table 3). For the more bulky bromo (**8c**), dichloro (**8d**) and *tert*-butyl (**8h**) derivatives only binding mode 2 was observed with the *tert*-butyl derivative the least energetically favourable. Two possible mode 1 orientations were observed for the 3-pyridyl derivative (**8j**) with indirect interaction with the haem via a water molecule either through imidiazole (S-value −5.32) or through one of the pyridine rings (S-value −6.58).

**Fig. 7:**
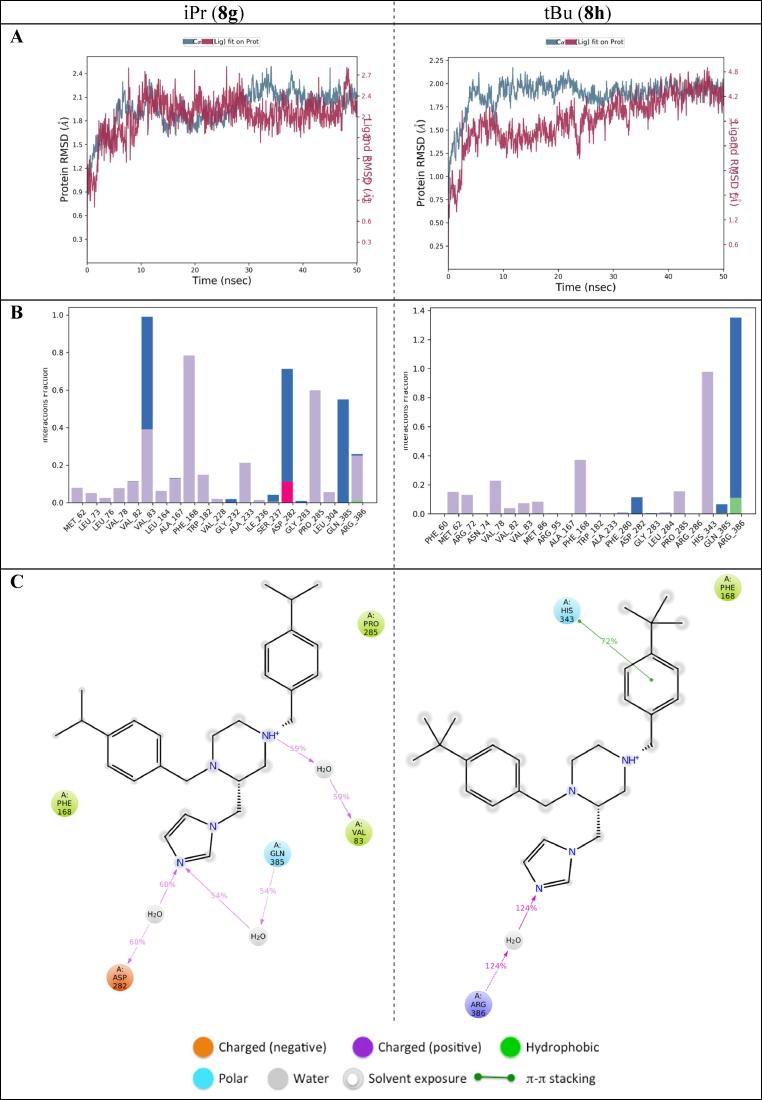
(A) Protein (blue) and ligand (red) root mean square of the CYP121A1-**8g** and **8h** complexes (**B**) Illustration of protein interactions with the ligand. The red column means ionic bond, green column a hydrogen bond, blue column water bridges, and a purple column a hydrophobic bond. The stacked bar charts are normalised over the course of the trajectory: for example, a value of 0.5 suggests that for 50% of the simulation time, the specific interaction is maintained (**C**) A schematic of detailed ligand atom interactions with the protein residues. Interactions that occur >30.0% of the simulation time in the selected trajectory (0.00 through 50.05 nsec), are shown (note: it is possible to have interactions with >100% as some residues may have multiple interactions of a single type with the same ligand atom e.g. the ARG side chain has four H-bond donors that can all hydrogen-bond to a single H-bond acceptor).

To investigate the binding modes further molecular dynamics simulations were run for 50 ns for the CYP121A1 and isopropyl derivative (**8g**) and *tert*-butyl derivative (**8h**) complexes using the Desmond programme of Maestro.^27^, ^28^ For the isopropyl (**8g**)-CYP121A1 complex, the RMSD changed from 1.2 Å at zero time to 2.1 Å at ∼10 ns; then the protein was equilibrated with no evident RMSD fluctuations observed and the ligand aligned. For the *tert*-butyl (**8h**)-CYP121A1 complex, equilibrium and ligand alignment required a longer run time with the RMSD changing from 0.8 Å at zero time to 2.0 Å at ∼30 ns. (Fig. 7A). Protein interactions with the ligand were checked throughout the simulation and interactions ordered by their type; specifically hydrogen bonds, hydrophobic, ionic, and water bridges (Fig. 7B). The isopropyl derivative (**8g**) formed more interactions over the course of the 50 ns simulation compared with the *tert*-butyl derivative (**8h**). As illustrated in Fig. 7C, water bridge interactions between the imidazole and Asp282 and Gln385, and the protonated piperazine and Val83 were significant for **8g** with additional hydrophobic interactions between the 4-iPr-phenyl rings and Phe168 and Pro285. For **8h** the major contributor was a water bridge interaction between the imidazole ring and Arg386, with additional binding interactions between one of the tBu-phenyl rings and His343 (π- π stacking) and Phe168 (hydrophobic) (Fig. 7C).

**Fig. 8:**
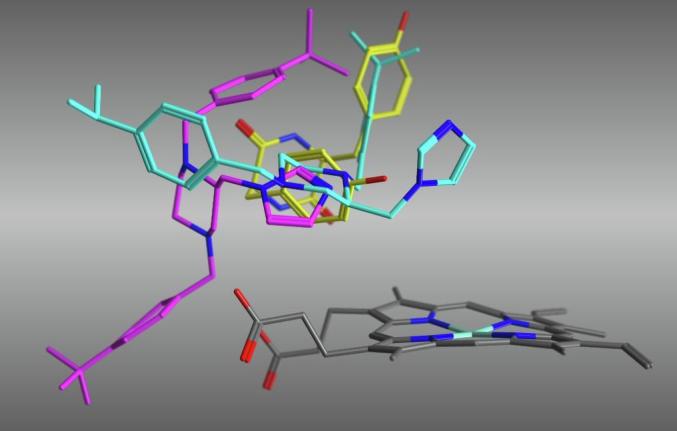
Positioning of the isopropyl derivative (**8g**, cyan) and *tert*-butyl derivative (**8h**, magenta) compared with cYY (yellow) after molecular dynamics simulation.

Sterically, the transition from an isopropyl (**8g**) to a *tert*-butyl (**8h**) would appear sufficient to hinder closer binding interaction. As shown in Fig. 8, the isopropyl derivative (**8g**) is able to take a position within the active site that is comparable (mode 1 binding) with the natural substrate cYY although one of the iPr-phenyl rings deviates from the cYY position through hydrophobic interaction with Pro285. However the *tert*-butyl derivative (**8h**) stabilises in the more open/flexible mode 2 conformation, with one of the tBu-phenyl rings deviating substantially, most likely owing to the reduction in conformational space within the binding pocket composed of Met62, Val82, Val83, Asn85 and Met86 (Fig. 6).

Both indirect binding modes 1 and 2 are possible and both would be effective in blocking the natural substrate cYY from binding at the active site. Crystallographic studies are needed to explore binding modes further.

## 3 Conclusions

With the exception of the pyridyl derivatives (**8i** and **8j**), all the new piperazine derivatives showed improved binding affinity to CYP121A1 and inhibitory activity against Mtb compared with the lead compound. Preliminary SAR for the described 1,4-dibenzyl-2-imidazol-1-yl-methylpiperazine derivatives would suggest that 4-substitution of the benzyl rings with either halides (**8a**–**8c**) or alkyl substitutents (**8f**–**8 h**) is beneficial for antimycobacterial activity with improved activity (MIC = 6.25 μg/mL) compared with clotrimazole (MIC = 20 μg/mL) (Table 1). Only one compound did not have substitution in the 4-position, namely the 3,5-dichloro derivative (**8d**), which had a reduced inhibitory activity compared with clotrimazole, however mono-substitutions in the 2- and 3-positions have not been investigated thus far and will be the focus of further studies to determine effect on binding and inhibitory activity. Two binding modes were observed from molecular modeling studies with the larger substituents (Br, 3,5-diCl, alkyl) showing a preference for the more open binding mode 2, while smaller substituents (F, Cl) showed a preference for the more compact binding mode 1 and more closely resemble the conformation of cYY (Table 2, Fig. 6). The alkyl derivatives (**8f**–**8h**) were optimal with respect to binding affinity (*K*_D_ = 3.7, 1.6 and 1.2 μM, respectively), with the ethyl (**8f**) and isopropyl derivatives (**8g**) inducing the most extensive Soret absorbance shifts (416.5 to 422.5/423.5 nm) (Table 1). However, binding affinity did not clearly correlate with MIC, for example, the 4-chloro derivative (**8b**) and the *tert*-butyl derivative (**8g**) showed similar MIC values (6.25 μg/mL) but had completely different binding affinity to CYP121A1 (26.8 ± 7.9 compared with 1.6 ± 0.1 μM), suggesting different ligation modes with either indirect or direct haem coordination.

These piperazine derivatives are useful compounds for further development with future studies focusing on replacement of one of the phenyl rings to optimise positioning within the binding pocket composed of Met62, Val82, Val83, Asn85 and Met86 identified from the computational studies as restricting optimal fit within the CYP121A1 active site.

## 4 Experimental section

### 4.1 General experimental

All chemicals, reagents and solvents were purchased from Sigma-Aldrich, Fisher Scientific, Alfa-Aesar, Fluka and Acros Chemicals. Whenever required, solvents were dried prior to use as described by the handbook Purification of Laboratory Chemicals and stored over 4 Å molecular sieves under nitrogen. Flash column chromatography was performed with silica gel (230–400 mesh) (Merck) and TLC was performed on pre-coated silica gel plates (Merck Kiesel gel 60_F254_, BDH). Melting points were determined on an electrothermal instrument (Gallenkamp), and are uncorrected. Compounds were visualised by irradiation with UV light at 254 nm and 365 nm or by using KMnO_4_ stain or vanillin stain followed by heating. NMR spectra were recorded on a Bruker AVANCE DPX500 spectrometer operating at 500,125 and 470 MHz for ^1^H, ^13^C and ^19^F NMR, respectively, and auto calibrated to the deuterated solvent reference peak. The assignments were made using one-dimensional (1D) and two-dimensional (2D) HSQC and COSY spectra. Chemical shifts are given in δ relative to tetramethylsilane (TMS); the coupling constants (*J*) are given in Hertz. TMS was used as an internal standard (*δ* = 0 ppm) for ^1^H NMR and CDCl_3_ served as an internal standard (*δ* = 77.0 ppm) for ^13^C NMR. Multiplicity is denoted as s (singlet), br s (broad singlet), d (doublet), dd (doublet of doublet), t (triplet), q (quartet), m (multiplet) or combinations thereof. High resolution mass spectra (HRMS) were determined at the EPSRC National Mass Spectrometry Facility at Swansea University and Medac Ltd., Brunel Science Centre, Surrey using ESI (Electrospray Ionisation). The following compounds were prepared as previously described: diimines **3a**-**3c**, **3e** and **3g**–**3j**;^29^, ^30^, ^31^, ^32^, ^33^, ^34^ diamines **4a**–**4c**, **4e**–**4f** and **4i**;^29^, ^30^, ^34^, ^35^ ethyl 1,4-bis(4-methoxybenzyl)piperazine-2-carboxylate (**5e**), (1,4-bis(4-methoxybenzyl)piperazin-2-yl)methanol (**6e**) and 2-(chloromethyl)-1,4-bis(4-methoxybenzyl)piperazine (**7e**).^18^ All compounds were>95% pure.

### 4.2 Chemistry

#### 4.2.1 General method for the preparation of diimines (3)

To a stirred solution of benzaldehyde (1) (2 m.eq.) in ethanol (1 mL/mmol of benzaldehyde), was added ethylenediamine (2) (1 m.eq.) dropwise. The reaction mixture was stirred for 6 h at room temperature, and the precipitated white coloured crystals were collected by filtration, washed with diethyl ether and dried to give the desired diimine.

##### 4.2.1.1 N,Ń-(Ethane-1,2-diyl)bis(1-(3,5-dichlorophenyl)methanimine) (**3d**)

Prepared from 3,5-dichlorobenzaldehyde (**1d**) (5 g, 28.6 mmol). Product obtained as a white solid, yield 5.10 g (96%). M.p. 130–132 °C. TLC (3:1 petroleum ether/EtOAc), R*f* = 0.84. ^1^H NMR (CDCl_3_): *δ* 8.34 (s, 2H, 2 × CH = N), 7.74 (d, *J* = 1.9 Hz, 4H, Ar), 7.71 (t, *J* = 1.9 Hz, 2H, *para*-Ar), 3.91 (s, 4H, 2 × CH_2_). ^13^C NMR (CDCl_3_): *δ* 161.3 (2 × CH = N), 135.7 (2 × C, Ar), 135.3 (4 × C, Ar), 129.9 (2 × CH, *para*-Ar), 129.3 (4 × CH, Ar), 61.2 (2 × CH_2_). [ESI-HRMS] calculated for C_16_H_13_Cl_4_N_2_: 372.9833 [M+H]^+^. Found: 372.9840 [M+H]^+^.

##### 4.2.1.2 N,N′-(Ethane-1,2-diyl)bis(1-(4-ethylphenyl)methanimine) (**3f**)

Prepared from 4-ethylbenzaldehyde (**1f**) (10 mL, 72.96 mmol). Product obtained as a white solid, yield 9.89 g (93%). M.p. 68–70 °C. TLC (4:1 petroleum ether/EtOAc), R*f* = 0.84. ^1^H NMR (CDCl_3_): *δ* 8.29 (s, 2H, 2 × CH = N), 7.62 (d, *J* = 8.1 Hz, 4H, Ar), 7.26 (d, *J* = 8.0 Hz, 4H, Ar), 3.84 (s, 4H, 2 × CH_2_), 2.62 (q, *J* = 7.6 Hz, 4H, 2 × CH_2_CH_3_), 1.17 (t, *J* = 7.6 Hz, 6H, 2 × CH_2_CH_3_). ^13^C NMR (DMSO-*d*_6_): *δ* 162.2 (2 × CH = N), 147.1 (2 × C, Ar), 134.2 (2 × C, Ar), 128.5 (4 × CH, Ar), 128.0 (4 × CH, Ar), 61.5 (2 × CH_2_), 28.5 (2 × CH_2_), 15.8 (2 × CH_3_). [ESI-HRMS] calculated for C_20_H_25_N_2_: 293.2018 [M+H]^+^. Found: 293.2033 [M+H]^+^.

#### 4.2.2 General method for the preparation of diamines (4)

To an ice-cooled stirred suspension of diimine (**3**) (1 m.eq.) in methanol (10 mL/mmol) was added sodium borohydride (4.6 m.eq.) in portions. The reaction mixture was stirred for 4 h at room temperature. After the reaction was complete, methanol was evaporated under vacuum and cold water (10 mL/mmol) was added to the remaining residue until cessation of effervescence. The mixture was extracted with EtOAc (10 mL/mmol), and the organic layer was washed with water (2 × 10 mL/mmol), brine (2 × 10 mL/mmol), dried (MgSO_4_) and evaporated under vacuum.

##### 4.2.2.1 N^1^,N^2^-Bis(3,5-dichlorobenzyl)ethane-1,2-diamine (**4d**)

Prepared from *N*,*N'*-(ethane-1,2-diyl)bis(1-(3,5-dichlorophenyl)methanimine) (**3d**) (5.0 g, 13.36 mmol). Product obtained as a white solid, yield 4.41 g (87%). M.p. 106–108 °C. TLC (2:1 petroleum ether/EtOAc), R*f* = 0.12. ^1^H NMR (CDCl_3_): *δ* 7.28 (m, 2H, *para*-Ar), 7.26 (m, 4H, Ar), 3.77 (s, 4H, 2 × CH_2_), 2.76 (s, 4H, 2 × CH_2_), 1.79 (br s, 2H, 2 × NH). ^13^C NMR (CDCl_3_): *δ* 143.6 (2 × C, Ar), 135.0 (4 × C, Ar), 127.2 (2 × CH, Ar), 126.5 (4 × CH, Ar), 52.8 (2 × CH_2_), 48.4 (2 × CH_2_). [ESI-HRMS] calculated for C_16_H_17_Cl_4_N_2_: 377.0146 [M+H]^+^. Found: 377.0159 [M+H]^+^.

##### 4.2.2.2 N^1^,N^2^-Bis(4-isopropylbenzyl)ethane-1,2-diamine (**4g**)

Prepared from *N*,*N'*-(ethane-1,2-diyl)bis(1-(4-isopropylphenyl)methanimine) (**3g**) (10.4 g, 32.76 mmol). Product obtained as a white solid, yield 9.89 g (93%). M.p. 58–60 °C. TLC (2:1 petroleum ether/EtOAc), R*f* = 0.51. ^1^H NMR (CDCl_3_): *δ* 7.22 (d, *J* = 8.2 Hz, 4H, Ar), 7.16 (d, *J* = 7.8 Hz, 4H, Ar), 3.62 (s, 4H, 2 × CH_2_), 2.85 (m, 2H, 2 × CH(CH_3_)_2_), 2.57 (s, 4H, 2 × CH_2_), 1.49 (br s, 2H, 2 × NH), 1.18 (d, *J* = 6.8 Hz, 12H, 4 × CH_3_). ^13^C NMR (DMSO-*d*_6_): *δ* 146.9 (2 × C, Ar), 138.9 (2 × C, Ar), 128.3 (4 × CH, Ar), 126.4 (4 × CH, Ar), 53.3 (2 × CH_2_), 48.9 (2 × CH_2_), 33.6 (2 × CH(CH_3_)_2_), 24.43 (4 × CH_3_). [ESI-HRMS] calculated for C_22_H_33_N_2_: 325.2638 [M+H]^+^. Found: 325.2643 [M+H]^+^.

##### 4.2.2.3 N^1^,N^2^-Bis(4-(*tert*-butyl)benzyl)ethane-1,2-diamine (**4h**)

Prepared from *N*,*N'*-(ethane-1,2-diyl)bis(1-(4-*tert*-butylylphenyl)methanimine) (**3h**) (7.0 g, 20.08 mmol). Product obtained as a colourless oil, yield 6.5 g (92%). TLC (2:1 petroleum ether/EtOAc), R*f* = 0.53. ^1^H NMR (DMSO-*d*_6_): *δ* 7.31 (d, *J* = 10.0 Hz, 4H, Ar), 7.21 (d, *J* = 10.0 Hz, 4H, Ar), 3.61 (s, 4H, 2 × CH_2_), 2.56 (s, 4H, 2 × CH_2_), 1.99 (br s, 2H, 2 × NH), 1.26 (s, 18H, 6 × CH_3_). ^13^C NMR (DMSO-*d*_6_): *δ* 149.2 (2 × C, Ar), 138.3 (2 × C, Ar), 128.1 (4 × CH, Ar), 125.2 (4 × CH, Ar), 53.1 (2 × CH_2_), 48.7 (2 × CH_2_), 34.6 (2 × C), 31.7 (6 × CH_3_). [ESI-HRMS] calculated for C_22_H_37_N_2_: 353.2951 [M+H]^+^. Found: 353.2948 [M+H]^+^.

##### 4.2.2.4 N^1^,N^2^-Bis(pyridin-3-yl methyl)ethane-1,2-diamine (**4j**)

Prepared from *N*,*N'*-(ethane-1,2-diyl)bis(1-(pyridine-3-yl)methanimine) (**3j**) (11.57 g, 48.57 mmol). Product obtained as a yellow oil, yield 3.7 g (31%). TLC (2:1 petroleum ether/EtOAc), R*f* = 0.12. ^1^H NMR (DMSO-*d*_6_): *δ* 8.84 (s, 2H, pyridine), 8.60 (m, 2H, pyridine), 8.15 (t, *J* = 5.8 Hz, 2H, pyridine), 7.41 (d, *J* = 5.7 Hz, 2H, pyridine), 3.85 (s, 4H, 2 × CH_2_), 3.71 (s, 4H, 2 × CH_2_), 2.46 (br s, 2H, 2 × NH). ^13^C NMR (DMSO-*d*_6_): *δ* 150.3 (2 × CH, pyridine), 148.8 (CH, pyridine), 148.7 (CH, pyridine), 136.7 (2 × CH, pyridine), 134.7 (C, pyridine), 133.9 (C, pyridine), 123.7 (2 × CH, pyridine), 53.6 (2 × CH_2_), 48.6 (2 × CH_2_). [ESI-HRMS] calculated for C_14_H_19_N_4_: 242.1531 [M+H]^+^. Found: 242.1536 [M+H]^+^.

#### 4.2.3 General method for the preparation of ethyl 1,4-bis(aryl)piperazine-2-carboxylates (5)

To a stirred solution of diamine (**4**) (1 m.eq., 2.84 g, 10.27 mmol) in anhydrous toluene at 80 °C (10 mL/mmol), was added triethylamine (2.5 m.eq.) and 2,3-dibromopropionic acid ethyl ester (1.05 m.eq.) dropwise, and the reaction mixture was heated at 80 °C overnight. The solvent was evaporated and the residue extracted with CH_2_Cl_2_ (10 mL/mmol), washed with saturated aqueous NaHCO_3_ (5 × 5 mL/mmol) and brine (3 × 5 mL/mmol). The organic layer was dried (MgSO_4_), concentrated under reduced pressure, and crude product was purified by gradient flash column chromatography.

##### 4.2.3.1 Ethyl 1,4-bis(4-fluorobenzyl)piperazine-2-carboxylate (**5a**)

Prepared from *N*^1^,*N*^2^-bis(4-fluorobenzyl)ethane-1,2-diamine (**4a**) (2.84 g, 10.27 mmol). The product was eluted with petroleum ether – EtOAc 87.5:12.5 v/v to give the product as a white solid, yield 2.49 g (65%). M.p. 46–48 °C. TLC (4:1 petroleum ether/EtOAc), R*f* = 0.82. ^1^H NMR (CDCl_3_): *δ* 7.29 (m, 4H, Ar), 6.99 (m, 4H, Ar), 4.19 (q, *J* = 7.0 Hz, 2H, CH_2_CH_3_), 3.89 (d, *J* = 13.3 Hz, 1H, H-7_a_), 3.58 (d, *J* = 13.0 Hz, 1H, H-7_b_), 3.53 (d, *J* = 13.2 Hz, 1H, H-8_a_), 3.40 (d, *J* = 13.2 Hz, 1H, H-8_b_), 3.30 (dd, *J* = 3.6, 5.5 Hz, 1H, H-2), 3.07 (m, 1H, H-3_a_), 2.76 (m, 1H, H-3_b_), 2.59–2.39 (m, 4H, H-6_a,b_, H-5_a,b_), 1.26 (t, *J* = 7.1 Hz, 3H, CH_2_CH_3_). ^13^C NMR (CDCl_3_): *δ* 172.0 (C=O), 163.0 (C, Ar), 162.9 (C, Ar), 161.1 (C, Ar), 161.0 (C, Ar), 130.5 (2 × CH, Ar), 130.4 (2 × CH, Ar), 130.3 (2 × CH, Ar), 130.2 (2 × CH, Ar), 62.6 (CH, piperazine), 61.8 (CH_2_CH_3_), 60.4 (CH_2_), 58.8 (CH_2_), 55.4 (CH_2_, piperazine), 53.0 (CH_2_, piperazine), 48.5 (CH_2_, piperazine), 14.3 (CH_2_CH_3_). ^19^F NMR (470 MHz, DMSO-*d*_6_): *δ* −115.99 (2 × C_6_H_5_F). [ESI-HRMS] calculated for C_21_H_25_F_2_N_2_O_2_: 375.1885 [M+H]^+^. Found: 375.1867 [M+H]^+^.

##### 4.2.3.2 Ethyl 1,4-bis(4-chlorobenzyl)piperazine-2-carboxylate (**5b**)

Prepared from *N*^1^,*N*^2^-bis(4-chlorobenzyl)ethane-1,2-diamine (**4b**) (4.00 g, 12.93 mmol). The product was eluted with petroleum ether – EtOAc 87.5:12.5 v/v to give the product as a white solid, yield 4.48 g (85%). M.p. 58–60 °C. TLC (4:1 petroleum ether/EtOAc), R*f* = 0.60. ^1^H NMR (CDCl_3_): *δ* 7.26 (m, 8H, Ar), 4.19 (q, *J* = 7.1 Hz, 2H, CH_2_CH_3_), 3.90 (d, *J* = 13.5 Hz, 1H, H-7_a_), 3.59 (d, *J* = 11.6 Hz, 1H, H-7_b_), 3.54 (d, *J* = 13.4 Hz, 1H, H-8_a_), 3.41 (d, *J* = 13.4 Hz, 1H, H-8_b_), 3.32 (m, 1H, H-2), 3.08 (m, 1H, H-3_a_), 2.77 (m, 1H, H-3_b_), 2.59 (m, 1H, H-6_a_), 2.53 (m, 1H, H-6_b_), 2.40 (m, 2H, H-5_a,b_), 1.26 (t, *J* = 7.1 Hz, 3H, CH_2_CH_3_). ^13^C NMR (CDCl_3_): *δ* 172.1 (C=O), 163.0 (2 × C, Ar), 161.1 (2 × C, Ar), 130.3 (4 × CH, Ar), 128.4 (4 × CH, Ar), 62.7 (CH, piperazine), 61.8 (CH_2_CH_3_), 60.4 (CH_2_), 58.8 (CH_2_), 55.4 (CH_2_, piperazine), 53.1 (CH_2_, piperazine), 48.4 (CH_2_, piperazine), 14.0 (CH_2_CH_3_). Anal. Calcd for C_21_H_24_Cl_2_N_2_O_2_ (407.3335): C, 61.92; H, 5.94; N, 6.87. Found: C, 61.66; H, 5.91; N, 6.84.

##### 4.2.3.3 Ethyl 1,4-bis(4-bromobenzyl)piperazine-2-carboxylate (**5c**)

Prepared from *N*^1^,*N*^2^-bis(4-bromobenzyl)ethane-1,2-diamine (**4c**) (9.35 g, 23.48 mmol). The product was eluted with petroleum ether – EtOAc 87.5:12.5 v/v to give the product as a white solid, yield 9.68 g (83%). M.p. 64–66 °C. TLC (4:1 petroleum ether/EtOAc), R*f* = 0.55. ^1^H NMR (CDCl_3_): *δ* 7.44 (m, 4H, Ar), 7.20 (m, 4H, Ar), 4.19 (q, *J* = 7.1 Hz, 2H, CH_2_CH_3_), 3.89 (d, *J* = 13.6 Hz, 1H, H-7_a_), 3.59 (d, *J* = 13.4 Hz, 1H, H-7_b_), 3.52 (d, *J* = 13.4 Hz, 1H, H-8_a_), 3.39 (d, *J* = 13.4 Hz, 1H, H-8_b_), 3.32 (dd, *J* = 3.4, 5.7 Hz, 1H, H-2), 3.08 (m, 1H, H-3_a_), 2.79 (m, 1H, H-3_b_), 2.55–2.38 (m, 4H, H-6_a,b_, H-5_a,b_), 1.26 (t, *J* = 7.1 Hz, 3H, CH_2_CH_3_). ^13^C NMR (CDCl_3_): *δ* 171.9 (C=O), 137.5 (2 × C, Ar), 137.1 (2 × C, Ar), 131.4 (2 × CH, Ar), 131.3 (2 × CH, Ar), 130.7 (2 × CH, Ar), 130.6 (2 × CH, Ar), 62.5 (CH, piperazine), 61.8 (CH_2_CH_3_), 60.5 (CH_2_), 58.9 (CH_2_), 55.4 (CH_2_, piperazine), 53.1 (CH_2_, piperazine), 48.4 (CH_2_, piperazine), 14.3 (CH_2_CH_3_). Anal. Calcd for C_21_H_24_Br_2_N_2_O_2_ (496.2355): C, 50.83; H, 4.84; N, 5.64. Found: C, 50.51; H, 4.80; N, 5.57.

##### 4.2.3.4 Ethyl 1,4-bis(3,5-dichlorobenzyl)piperazine-2-carboxylate (**5d**)

Prepared from *N*^1^,*N*^2^-bis(3,5-dichlorobenzyl)ethane-1,2-diamine (**4d**) (6.0 g, 16.87 mmol). The product was eluted with petroleum ether – EtOAc 9:1 v/v to give the product as a buff solid, yield 4.46 g (71%). M.p. 80–84 °C. TLC (4:1 petroleum ether/EtOAc), R*f* = 0.85. ^1^H NMR (DMSO-*d*_6_): *δ* 7.79 (m, 2H, *para*-Ar), 7.36 (m, 4H, Ar), 4.09 (q, *J* = 7.1 Hz, 2H, CH_2_CH_3_), 3.87 (d, *J* = 13.7 Hz, 1H, H-7_a_), 3.72 (d, *J* = 14.0 Hz, 1H, H-7_b_), 3.61 (d, *J* = 14.0 Hz, 1H, H-8_a_), 3.47 (d, *J* = 14.0 Hz, 1H, H-8_b_), 3.38 (m, 1H, H-2), 3.36 (m, 1H, H-3_a_), 3.03 (m, 1H, H-3_b_), 2.78 (m, 1H, H-6_a_), 2.62 (m, 1H, H-6_b_), 2.44–2.30 (m, 2H, H-5_a,b_), 1.15 (t, *J* = 7.1 Hz, 3H, CH_2_CH_3_). ^13^C NMR (DMSO-*d*_6_): *δ* 171.7 (C=O), 144.4 (2 × C, Ar), 143.4 (2 × C, Ar), 134.4 (C, Ar), 134.3 (C, Ar), 127.5 (2 × CH, Ar), 127.3 (2 × CH, Ar), 127.1 (CH, Ar), 126.9 (CH, Ar), 62.5 (CH, piperazine), 60.4 (CH_2_CH_3_), 60.4 (CH_2_), 57.8 (CH_2_), 55.2 (CH_2_, piperazine), 53.3 (CH_2_, piperazine), 48.5 (CH_2_, piperazine), 14.5 (CH_2_CH_3_). Anal. Calcd for C_21_H_22_Cl_4_N_2_O_2_ (476.2236): C, 52.96; H, 4.66; N, 5.88. Found: C, 52.69; H, 4.57; N, 5.81.

##### 4.2.3.5 Ethyl 1,4-bis(4-ethylbenzyl)piperazine-2-carboxylate (**5f**)

Prepared from *N*^1^,*N*^2^-bis(4-ethylbenzyl)ethane-1,2-diamine (**4f**) (9.0 g, 30.35 mmol). The product was eluted with petroleum ether – EtOAc 9:1 v/v to give the product as a colourless oil, yield 8.8 g (74%). TLC (95:5 CH_2_Cl_2_/MeOH), R*f* = 0.97. ^1^H NMR (DMSO-*d*_6_): *δ* 7.15 (m, 8H, Ar), 4.06 (q, *J* = 7.7 Hz, 2H, CH_2_CH_3_), 3.79 (d, *J* = 13.4 Hz, 1H, H-7_a_), 3.57 (d, *J* = 13.3 Hz, 1H, H-7_b_), 3.48 (d, *J* = 13.2 Hz, 1H, H-8_a_), 3.29 (m, 2H, H-8_b_, H-2), 2.98 (m, 1H, H-3_a_), 2.69 (m, 1H, H-3_b_), 2.56 (q, *J* = 7.5 Hz, 4H, 2 × CH_2_CH_3_), 2.40 (m, 4H, H-6_a,b_, H-5_a,b_), 1.15 (m, 9H, 3 × CH_2_CH_3_). ^13^C NMR (DMSO-*d*_6_): *δ* 171.7 (C=O), 142.8 (2 × C, Ar), 135.6 (2 × C, Ar), 129.0 (4 × CH, Ar), 128.0 (4 × CH, Ar), 64.7 (CH, piperazine), 63.0 (CH_2_CH_3_), 61.9 (CH_2_), 60.7 (CH_2_), 58.8 (CH_2_, piperazine), 55.4 (CH_2_, piperazine), 53.3 (CH_2_, piperazine), 28.3 (2 × CH_2_CH_3_), 16.1 (2 × CH_2_CH_3_), 14.5 (CH_2_CH_3_). [ESI-HRMS] calculated for C_25_H_35_N_2_O_2_: 395.2699 [M+H]^+^. Found: 395.2692 [M+H]^+^.

##### 4.2.3.6 Ethyl 1,4-bis(4-isopropylbenzyl)piperazine-2-carboxylate (**5g**)

Prepared from *N*^1^,*N*^2^-bis(4-isopropylbenzyl)ethane-1,2-diamine (**4f**) (9.0 g, 27.73 mmol). The product was eluted with petroleum ether – EtOAc 9:1 v/v to give the product as a colourless oil, yield 10.0 g (85%). TLC (95:5 CH_2_Cl_2_/MeOH), R*f* = 0.98. ^1^H NMR (DMSO-*d*_6_): *δ* 7.17 (m, 8H, Ar), 4.08 (q, *J* = 7.8 Hz, 2H, CH_2_CH_3_), 3.79 (d, *J* = 13.3 Hz, 1H, H-7_a_), 3.57 (d, *J* = 13.2 Hz, 1H, H-7_b_), 3.48 (d, *J* = 13.1 Hz, 1H, H-8_a_), 3.27 (m, 2H, H-8_b_, H-2), 2.99 (m, 1H, H-3_a_), 2.84 (sept, 2H, *J* = 6.8 Hz, 2 × CH(CH_3_)_2_), 2.70 (m, 1H, H-3_b_), 2.47–2.30 (m, 4H, H-6_a,b_, H-5_a,b_), 1.17 (d, *J* = 7.0 Hz, 12H, 4 × CH_3_), 1.11 (t, *J* = 7.2 Hz, 3H, CH_2_CH_3_). ^13^C NMR (DMSO-*d*_6_): *δ* 171.7 (C=O), 147.4 (2 × C, Ar), 136.3 (C, Ar), 135.8 (C, Ar), 129.0 (2 × CH, Ar), 128.9 (2 × CH, Ar), 126.5 (2 × CH, Ar), 126.4 (2 × CH, Ar), 65.4 (CH, piperazine), 61.9 (CH_2_CH_3_), 60.2 (CH_2_), 60.1 (CH_2_), 58.8 (CH_2_, piperazine), 55.4 (CH_2_, piperazine), 53.4 (CH_2_, piperazine), 33.6 (2 × CH(CH_3_)_2_), 24.4 (4 × CH_3_), 14.5 (CH_2_CH_3_). [ESI-HRMS] calculated for C_27_H_39_N_2_O_2_: 423.3012 [M+H]^+^. Found: 423.3016 [M+H]^+^.

##### 4.2.3.7 Ethyl 1,4-bis(4-(*tert*-butyl)benzyl)piperazine-2-carboxylate (**5h**)

Prepared from *N*^1^,*N*^2^-bis(4-*tert-*butylbenzyl)ethane-1,2-diamine (**4h**) (6.0 g, 17.01 mmol). The product was eluted with petroleum ether – EtOAc 92.5:7.5 v/v to give the product as a white solid, yield 5.0 g (65%). M.p. 82–84 °C. TLC (5:1 petroleum ether/EtOAc), R*f* = 0.83. ^1^H NMR (CDCl_3_): *δ* 7.31 (m, 4H, Ar), 7.18 (m, 4H, Ar), 4.06 (q, *J* = 7.1 Hz, 2H, CH_2_CH_3_), 3.80 (d, *J* = 13.1 Hz, 1H, H-7_a_), 3.54 (d, *J* = 13.2 Hz, 1H, H-7_b_), 3.39 (m, 2H, H-8_a,b_), 3.30 (dd, *J* = 3.6, 5.6 Hz, 1H, H-2), 2.99 (m, 1H, H-3_a_), 2.72 (m, 1H, H-3_b_), 2.41 (m, 4H, H-6_a,b_, H-5_a,b_), 1.25 (s, 18H, 6 × CH_3_), 1.11 (t, *J* = 6.7 Hz, 3H, CH_2_CH_3_). ^13^C NMR (CDCl_3_): *δ* 171.5 (C=O), 135.8 (2 × C, Ar), 132.2 (2 × C, Ar), 128.8 (4 × CH, Ar), 125.4 (4 × CH, Ar), 64.1 (CH, piperazine), 63.0 (CH_2_CH_3_), 61.8 (CH_2_), 60.3 (CH_2_), 58.6 (CH_2_, piperazine), 53.2 (CH_2_, piperazine), 47.9 (CH_2_, piperazine), 34.6 (2 × C(CH_3_)_3_), 31.6 (6 × CH_3_), 14.5 (CH_2_CH_3_). [ESI-HRMS] calculated for C_29_H_43_N_2_O_2_: 451.3324 [M+H]^+^. Found: 451.3327 [M+H]^+^.

##### 4.2.3.8 Ethyl 1,4-bis(pyridin-4-ylmethyl)piperazine-2-carboxylate (**5i**)

Prepared from *N*^1^,*N*^2^-bis(pyridin-4-yl)ethane-1,2-diamine (**4i**) (3.66 g, 15.10 mmol). The product was eluted with CH_2_Cl_2_-MeOH-Et_3_N 97.5:1.5:1 v/v/v to give the product as a brown oil, yield 3.36 g (65%). TLC (95:5 CH_2_Cl_2_/MeOH), R*f* = 0.32. ^1^H NMR (DMSO-*d*_6_): *δ* 8.50 (m, 4H, Ar), 7.30 (m, 4H, Ar), 4.10 (q, *J* = 7.6 Hz, 2H, CH_2_CH_3_), 3.90 (d, *J* = 13.0 Hz, 1H, H-7_a_), 3.74 (d, *J* = 13.7 Hz, 1H, H-7_b_), 3.61 (d, *J* = 13.4 Hz, 1H, H-8_a_), 3.46 (d, *J* = 13.4 Hz, 1H, H-8_b_), 3.40 (dd, *J* = 3.6, 5.9 Hz, 1H, H-2), 3.04 (m, 1H, H-3_a_), 2.81 (m, 1H, H-3_b_), 2.58 (m, 1H, H-6_a_), 2.45 (m, 1H, H-6_b_), 2.36 (m, 2H, H-5_a,b_), 1.15 (t, *J* = 7.0 Hz, 3H, CH_2_CH_3_). ^13^C NMR (DMSO-*d*_6_): *δ* 171.6 (C=O), 150.0 (4 × CH, Ar), 148.7 (C, Ar), 147.7 (C, Ar), 124.0 (2 × CH, Ar), 123.8 (2 × CH, Ar), 62.0 (CH, piperazine), 60.7 (CH_2_CH_3_), 60.4 (CH_2_), 57.8 (CH_2_), 55.4 (CH_2_, piperazine), 53.4 (CH_2_, piperazine), 52.5 (CH_2_, piperazine), 14.6 (CH_2_CH_3_). [ESI-HRMS] calculated for C_19_H_25_N_4_O_2_: 341.1899 [M+H]^+^. Found: 341.1910 [M+H]^+^.

##### 4.2.3.9 Ethyl 1,4-bis(pyridin-3-ylmethyl)piperazine-2-carboxylate (**5j**)

Prepared from *N*^1^,*N*^2^-bis(pyridin-3-yl)ethane-1,2-diamine (**4j**) (3.50 g, 14.44 mmol). The product was eluted with CH_2_Cl_2_-MeOH-Et_3_N 96.5:2.5:1 v/v/v to give the product as a brown oil, yield 2.30 g (47%). TLC (95:5 CH_2_Cl_2_/MeOH), R*f* = 0.31. ^1^H NMR (DMSO-*d*_6_): *δ* 8.47 (s, 2H, pyridine), 8.46 (m, 2H, pyridine), 7.67 (t, *J* = 7.7 Hz, 2H, pyridine), 7.35 (d, *J* = 5.6 Hz, 2H, pyridine), 4.08 (q, *J* = 7.8 Hz, 2H, CH_2_CH_3_), 3.88 (d, *J* = 14.0 Hz, 1H, H-7_a_), 3.70 (d, *J* = 13.8 Hz, 1H, H-7_b_), 3.60 (d, *J* = 13.5 Hz, 1H, H-8_a_), 3.38 (m, 2H, H-8_b_, H-2), 3.00 (m, 1H, H-3_a_), 2.75 (m, 1H, H-3_b_), 2.54 (m, 1H, H-6_a_), 2.42 (m, 1H, H-6_b_), 2.34 (m, 2H, H-5_a,b_), 1.13 (t, *J* = 7.7 Hz, 3H, CH_2_CH_3_). ^13^C NMR (DMSO-*d*_6_): *δ* 171.6 (C=O), 150.4 (CH, pyridine), 150.2 (CH, pyridine), 148.8 (CH, pyridine), 148.7 (CH, pyridine), 136.9 (CH, pyridine), 136.6 (CH, pyridine), 134.7 (C, pyridine), 133.9 (C, pyridine), 123.9 (CH, pyridine), 123.8 (CH, pyridine), 62.3 (CH, piperazine), 60.4 (CH_2_CH_3_), 59.1 (CH_2_), 56.3 (CH_2_), 56.0 (CH_2_, piperazine), 55.2 (CH_2_, piperazine), 53.2 (CH_2_, piperazine), 14.5 (CH_2_CH_3_). [ESI-HRMS] calculated for C_19_H_25_N_4_O_2_: 341.1899 [M+H]^+^. Found: 341.1903 [M+H]^+^.

#### 4.2.4 General method for the preparation of alcohols (6)

To an ice-cooled solution of ethyl carboxylate (**5**) (1 m.eq.) in dry THF (3 mL/mmol) was added LiAlH_4_ (1 M in THF, 1.5 m.eq.) dropwise over 25 min. The reaction was then stirred at room temperature overnight, then cooled in an ice-bath and carefully quenched with H_2_O until cessation of effervescence. The reaction mixture was extracted with EtOAc (2 × 10 mL/mmol), then the combined organic layers washed with H_2_O (3 × 10 mL/mmol), dried (MgSO_4_) and concentrated under reduced pressure. The crude product was purified by gradient flash column chromatography.

##### 4.2.4.1 (1,4-Bis(4-fluorobenzyl)piperazin-2-yl)methanol (**6a**)

Prepared from ethyl 1,4-bis(4-fluorobenzyl)piperazine-2-carboxylate (**5a**) (1.2 g, 3.20 mmol). The product was eluted with CH_2_Cl_2_-MeOH 95:5 v/v to give the product as a yellow oil, yield 0.86 g (81%). TLC (95:5 CH_2_Cl_2_/MeOH), R*f* = 0.55. ^1^H NMR (CDCl_3_): *δ* 7.30 (m, 8H, Ar), 3.61 (m, 2H, H-9_a,b_), 3.48 (m, 2H, H-7_a,b_), 2.96 (m, 2H, H-8_a,b_), 2.67 (m, 3H, H-2, H-3_a,b_), 2.59 (m, 1H, H-6_a_), 2.53–2.45 (m, 2H, H-6_b_, H-5_a_), 2.37 (m, 1H, H-5_b_). ^13^C NMR (CDCl_3_): *δ* 163.1 (C, Ar), 161.1 (C, Ar), 138.4 (C, Ar), 137.5 (C, Ar), 128.9 (4 × CH, Ar), 128.4 (4 × CH, Ar), 62.3 (CH_2_), 58.7 (CH, piperazine), 58.0 (CH_2_), 57.2 (CH_2_), 55.9 (CH_2_, piperazine), 52.4 (CH_2_, piperazine), 49.9 (CH_2_, piperazine). ^19^F NMR (CDCl_3_): *δ* −116.00 (2 × C_6_H_5_F). [ESI-HRMS] calculated for C_19_H_23_F_2_N_2_O: 333.1807 [M+H]^+^. Found: 333.1823 [M+H]^+^.

##### 4.2.4.2 (1,4-Bis(4-chlorobenzyl)piperazin-2-yl)methanol (**6b**)

Prepared from ethyl 1,4-bis(4-chlorobenzyl)piperazine-2-carboxylate (**5b**) (1.0 g, 2.45 mmol). The product was eluted with CH_2_Cl_2_-MeOH 97:3 v/v to give the product as a colourless oil, yield 0.52 g (59%). TLC (95:5 CH_2_Cl_2_/MeOH), R*f* = 0.59. ^1^H NMR (DMSO-*d*_6_): *δ* 7.30 (m, 8H, Ar), 4.50 (t, *J* = 5.1 Hz, 1H, OH), 3.69 (m, 2H, H-9_a,b_), 3.43 (m, 2H, H-7_a,b_), 3.38 (d, *J* = 13.6 Hz, 1H, H-8_a_), 3.27 (d, *J* = 13.6 Hz, 1H, H-8_b_), 2.73 (m, 1H, H-2), 2.59 (m, 1H, H-3_a_), 2.48 (m, 1H, H-3_b_), 2.41 (m, 1H, H-6_a_), 2.18–2.02 (m, 3H, H-6_b_, H-5_a,b_). ^13^C NMR (DMSO-*d*_6_): *δ* 138.9 (C, Ar), 137.8 (C, Ar), 131.9 (C, Ar), 131.6 (C, Ar), 130.9 (2 × CH, Ar), 130.8 (2 × CH, Ar), 128.6 (2 × CH, Ar), 128.5 (2 × CH, Ar), 62.7 (CH_2_), 61.8 (CH, piperazine), 61.6 (CH_2_), 58.2 (CH_2_), 57.3 (CH_2_, piperazine), 52.9 (CH_2_, piperazine), 51.1 (CH_2_, piperazine). [ESI-HRMS] calculated for C_19_H_23_Cl_2_N_2_O: 365.1187 [M+H]^+^. Found: 365.1190 [M+H]^+^.

##### 4.2.4.3 (1,4-Bis(4-bromobenzyl)piperazin-2-yl)methanol (**6c**)

Prepared from ethyl 1,4-bis(4-bromobenzyl)piperazine-2-carboxylate (**5c**) (1.20 g, 2.41 mmol). The product was eluted with CH_2_Cl_2_-MeOH 97.5:2.5 v/v to give the product as a white solid, yield 0.89 g (81%). M.p. 88–90 °C. TLC (95:5 CH_2_Cl_2_/MeOH), R*f* = 0.63. ^1^H NMR (DMSO-*d*_6_): *δ* 7.49 (m, 4H, Ar), 7.25 (m, 4H, Ar), 4.54 (t, *J* = 5.1 Hz, 1H, OH), 3.66 (m, 2H, H-9_a,b_), 3.42 (m, 2H, H-7_a,b_), 3.38 (d, *J* = 13.8 Hz, 1H, H-8_a_), 3.26 (d, *J* = 13.9 Hz, 1H, H-8_b_), 2.69 (m, 1H, H-2), 2.56 (m, 1H, H-3_a_), 2.47 (m, 1H, H-3_b_), 2.40 (m, 1H, H-6_a_), 2.17–2.02 (m, 3H, H-6_b_, H-5_a,b_). ^13^C NMR (DMSO-*d*_6_): *δ* 138.2 (2 × C, Ar), 131.5 (4 × CH, Ar), 131.2 (4 × CH, Ar), 120.3 (2 × C, Ar), 62.7 (CH_2_), 61.8 (CH, piperazine), 61.6 (CH_2_), 58.1 (CH_2_), 56.4 (CH_2_, piperazine), 53.0 (CH_2_, piperazine), 51.1 (CH_2_, piperazine). [ESI-HRMS] calculated for C_19_H_23_Br_2_N_2_O: 453.0177 [M+H]^+^. Found: 453.0163 [M+H]^+^.

##### 4.2.4.4 (1,4-Bis(3,5-dichlorobenzyl)piperazin-2-yl)methanol (**6d**)

Prepared from ethyl 1,4-bis(3,5-dichlorobenzyl)piperazine-2-carboxylate (**5d**) (1.5 g, 3.14 mmol). The product was eluted with petroleum ether – EtOAc 7:3 v/v v/v to give the product as a yellow oil, yield 0.75 g (56%). TLC (1:1 petroleum ether/EtOAc), R*f* = 0.31. ^1^H NMR (DMSO-*d*_6_): *δ* 7.46 (t, *J* = 1.7 Hz, 1H, *para*-Ar), 7.43 (t, *J* = 1.7 Hz, 1H, *para*-Ar), 7.36 (d, *J* = 1.7 Hz, 2H, Ar), 7.33 (d, *J* = 1.7 Hz, 2H, Ar), 4.60 (t, *J* = 5.1 Hz, 1H, OH), 3.63 (m, 2H, H-9_a,b_), 3.45 (m, 2H, H-7_a,b_), 3.37 (m, 2H, H-8_a,b_), 2.68 (m, 1H, H-2), 2.58 (m, 1H, H-3_a_), 2.48 (m, 1H, H-3_b_), 2.43 (m, 1H, H-6_a_), 2.21–2.07 (m, 3H, H-6_b_, H-5_a,b_). ^13^C NMR (DMSO-*d*_6_): *δ* 142.5 (2 × C, Ar), 141.3 (2 × C, Ar), 133.4 (2 × C, Ar), 130.5 (CH, Ar), 130.4 (CH, Ar), 128.6 (CH, Ar), 128.5 (CH, Ar), 127.9 (CH, Ar), 127.7 (CH, Ar), 61.7 (CH_2_), 61.6 (CH, piperazine), 61.5 (CH_2_), 57.4 (CH_2_), 56.9 (CH_2_, piperazine), 52.8 (CH_2_, piperazine), 51.1 (CH_2_, piperazine). [ESI-HRMS] calculated for C_19_H_21_Cl_4_N_2_O: 433.0408 [M+H]^+^. Found: 433.0399 [M+H]^+^.

##### 4.2.4.5 (1,4-Bis(4-ethylbenzyl)piperazin-2-yl)methanol (**6f**)

Prepared from ethyl 1,4-bis(4-ethylbenzyl)piperazine-2-carboxylate (**5f**) (2.0 g, 5.06 mmol). The product was eluted with CH_2_Cl_2_-MeOH 97:3 v/v to give the product as a buff solid, yield 1.48 g (83%). M.p. 40–42 °C. TLC (95:5 CH_2_Cl_2_/MeOH), R*f* = 0.65. ^1^H NMR (DMSO-*d*_6_): *δ* 7.18 (m, 8H, Ar), 4.52 (br s, 1H, OH), 3.70 (m, 2H, H-9_a,b_), 3.38 (m, 2H, H-7_a,b_), 3.22 (d, *J* = 13.7 Hz, 1H, H-8_a_), 2.73 (d, *J* = 13.4 Hz, 1H, H-8_b_), 2.57 (q, *J* = 7.4 Hz, 4H, 2 × CH_2_CH_3_), 2.38 (m, 1H, H-2), 2.14–2.02 (m, 6H, H-3_a,b_, H-6_a,b_, H-5_a,b_), 1.16 (t, *J* = 7.4 Hz, 6H, 2 × CH_3_). ^13^C NMR (DMSO-*d*_6_): *δ* 142.7 (C, Ar), 142.4 (C, Ar), 136.8 (C, Ar), 135.9 (C, Ar), 129.2 (2 × CH, Ar), 129.1 (2 × CH, Ar), 128.0 (2 × CH, Ar), 127.9 (2 × CH, Ar), 62.5 (CH_2_), 61.7 (CH, piperazine), 60.2 (CH_2_), 57.7 (CH_2_), 56.4 (CH_2_, piperazine), 53.0 (CH_2_, piperazine), 51.1 (CH_2_, piperazine), 28.3 (2 × CH_2_CH_3_), 16.1 (2 × CH_2_CH_3_). [ESI-HRMS] calculated for C_23_H_33_N_2_O: 353.2587 [M+H]^+^. Found: 353.2610 [M+H]^+^.

##### 4.2.4.6 (1,4-Bis(4-isopropylbenzyl)piperazin-2-yl)methanol (**6g**)

Prepared from ethyl 1,4-bis(4-isopropylbenzyl)piperazine-2-carboxylate (**5 g**) (2.50 g, 5.91 mmol). The product was eluted with CH_2_Cl_2_-MeOH 97:3 v/v to give the product as a buff solid, yield 1.91 g (85%). M.p. 68–70 °C. TLC (95:5 CH_2_Cl_2_/MeOH), R*f* = 0.68. ^1^H NMR (DMSO-*d*_6_): *δ* 7.17 (m, 8H, Ar), 4.53 (br s, 1H, OH), 3.71 (m, 2H, H-9_a,b_), 3.40 (d, *J* = 13.1 Hz, 1H, H-7_a_), 3.37 (d, *J* = 13.1 Hz, 1H, H-7_b_), 3.21 (d, *J* = 13.8 Hz, 1H, H-8_a_), 2.85 (sept, *J* = 3.2 Hz, 2H, 2 × CH(CH_3_)_2_), 2.74 (d, *J* = 13.2 Hz, 1H, H-8_b_), 2.58 (m, 1H, H-2), 2.47–2.39 (m, 2H, H-3_a,b_), 2.15–2.01 (m, 4H, H-6_a,b_, H-5_a,b_), 1.19 (d, *J* = 1.9 Hz, 6H, 2 × CH_3_), 1.17 (d, *J* = 1.8 Hz, 6H, 2 × CH_3_). ^13^C NMR (DMSO-*d*_6_): *δ* 137.0 (2 × C, Ar), 136.0 (2 × C, Ar), 129.2 (2 × CH, Ar), 129.0 (2 × CH, Ar), 126.5 (2 × CH, Ar), 126.4 (2 × CH, Ar), 62.5 (CH_2_), 61.8 (CH, piperazine), 60.2 (CH_2_), 57.9 (CH_2_), 56.5 (CH_2_, piperazine), 53.1 (CH_2_, piperazine), 51.2 (CH_2_, piperazine), 33.6 (2 × CH(CH_3_)_2_), 24.4 (4 × CH_3_). [ESI-HRMS] calculated for C_25_H_37_N_2_O: 381.2900 [M+H]^+^. Found: 381.2890 [M+H]^+^.

##### 4.2.4.7 (1,4-Bis(4-(*tert*-butyl)benzyl)piperazin-2-yl)methanol (**6h**)

Prepared from ethyl 1,4-bis(4-*tert*butylylbenzyl)piperazine-2-carboxylate (**5 h**) (5 g, 11.09 mmol). The product was eluted with CH_2_Cl_2_-MeOH 97:3 v/v to give the product as a white solid, yield 4.12 g (91%). M.p. 92–94 °C. TLC (95:5 CH_2_Cl_2_/MeOH), R*f* = 0.71. ^1^H NMR (DMSO-*d*_6_): *δ* 7.30 (m, 4H, Ar), 7.21 (m, 4H, Ar), 4.55 (br s, 1H, OH), 3.70 (m, 2H, H-9_a,b_), 3.41 (d, *J* = 13.2 Hz, 1H, H-7_a_), 3.23 (d, *J* = 13.1 Hz, 1H, H-7_b_), 2.77 (d, *J* = 12.6 Hz, 1H, H-8_a_), 2.58 (d, *J* = 12.7 Hz, 1H, H-8_b_), 2.44 (m, 3H, H-2, H-3_a,b_), 2.41 (m, 2H, H-6_a,b_), 2.06 (m, 2H, H-5_a,b_), 1.27 (s, 9H, 3 × CH_3_), 1.26 (s, 9H, 3 × CH_3_). ^13^C NMR (DMSO-*d*_6_): *δ* 149.5 (C, Ar), 149.2 (C, Ar), 136.6 (C, Ar), 135.6 (C, Ar), 128.9 (2 × CH, Ar), 128.8 (2 × CH, Ar), 125.2 (2 × CH, Ar), 125.1 (2 × CH, Ar), 62.5 (CH_2_), 61.8 (CH, piperazine), 60.2 (CH_2_), 57.9 (CH_2_), 56.6 (CH_2_, piperazine), 53.1 (CH_2_, piperazine), 51.2 (CH_2_, piperazine), 34.5 (2 × C(CH_3_)_3_), 31.6 (6 × CH_3_). [ESI-HRMS] calculated for C_27_H_41_N_2_O: 409.3213 [M+H]^+^. Found: 409.3215 [M+H]^+^.

##### 4.2.4.8 (1,4-Bis(pyridine-4-yl)piperazin-2-yl)methanol (**6i**)

Prepared from ethyl 1,4-bis(pyridin-4-yl)piperazine-2-carboxylate (**5i**) (0.96 g, 2.82 mmol). The product was eluted with CH_2_Cl_2_-MeOH-Et_3_N 89:10:1 v/v/v to give the product as a brown oil, yield 0.60 g (71%). TLC (95:5 CH_2_Cl_2_/MeOH), R*f* = 0.25. ^1^H NMR (DMSO-*d*_6_): *δ* 8.49 (m, 4H, Ar), 7.32 (m, 4H, Ar), 4.58 (t, *J* = 5.2 Hz, 1H, OH), 3.65 (m, 2H, H-9_a,b_), 3.49 (m, 2H, H-7_a,b_), 3.39 (m, 2H, H-8_a,b_), 2.70 (m, 1H, H-2), 2.61 (m, 1H, H-3_a_), 2.46 (m, 1H, H-3_b_), 2.26–2.16 (m, 3H, H-6_a,b_, H-5_a_), 2.10 (m, 1H, H-5_b_). ^13^C NMR (DMSO-*d*_6_): *δ* 136.8 (2 × C, pyridine), 129.3 (4 × CH, pyridine), 126.7 (4 × CH, pyridine), 62.5 (CH_2_), 61.6 (CH, piperazine), 60.1 (CH_2_), 57.5 (CH_2_), 56.4 (CH_2_, piperazine), 52.9(CH_2_, piperazine), 51.4 (CH_2_, piperazine). [ESI-HRMS] calculated for C_17_H_23_N_4_O: 299.1872 [M+H]^+^. Found: 299.1882 [M+H]^+^.

##### 4.2.4.9 (1,4-Bis(pyridine-3-yl)piperazin-2-yl)methanol (**6j**)

Prepared from ethyl 1,4-bis(pyridin-3-yl)piperazine-2-carboxylate (**5j**) (1.10 g, 3.23 mmol). The product was eluted with CH_2_Cl_2_-MeOH-Et_3_N 89:10:1 v/v/v to give the product as a brown oil, yield 0.68 g (71%). TLC (95:5 CH_2_Cl_2_/MeOH), R*f* = 0.24. ^1^H NMR (DMSO-*d*_6_): *δ* 8.49 (s, 1H, pyridine), 8.47 (s, 1H, pyridine), 8.45 (m, 2H, pyridine), 7.70 (t, *J* = 7.7 Hz, 2H, pyridine), 7.34 (d, *J* = 5.6 Hz, 2H, pyridine), 4.57 (t, *J* = 5.2 Hz, 1H, OH), 3.70 (m, 2H, H-9_a,b_), 3.48 (m, 2H, H-7_a,b_), 3.41 (m, 2H, H-8_a,b_), 2.70 (m, 1H, H-2), 2.57 (m, 1H, H-3_a_), 2.41 (m, 1H, H-3_b_), 2.20–2.04 (m, 4H, H-6_a,b_, H-5_a_,_b_). ^13^C NMR (DMSO-*d*_6_): *δ* 150.1 (CH, pyridine), 149.8 (CH, pyridine), 148.5 (CH, pyridine), 147.6 (CH, pyridine), 136.8 (2 × CH, pyridine), 134.3 (2 × C, pyridine), 121.9 (2 × CH, pyridine), 62.4 (CH_2_), 61.2 (CH, piperazine), 59.7 (CH_2_), 56.3 (CH_2_), 53.6 (CH_2_, piperazine), 51.3 (CH_2_, piperazine), 49.8 (CH_2_, piperazine). [ESI-HRMS] calculated for C_17_H_23_N_4_O: 299.1872 [M+H]^+^. Found: 299.1879 [M+H]^+^.

#### 4.2.5 General method for the preparation of chlorides (7)

To an ice-cooled solution of alcohol (**6**) (1 m.eq.) in dry CH_2_Cl_2_ (5 mL/mmol) was added thionyl chloride (10 m.eq.) dropwise over 25 min. The reaction was stirred at room temperature for 48 h then cooled in an ice-bath and carefully quenched with saturated aqueous NaHCO_3_ in portions until slightly basic (pH 8.0). The organic layer was separated, washed with brine (3 × 10 mL/mmol), H_2_O (2 × 10 mL/mmol), dried (MgSO_4_) and evaporated under reduced pressure to give the chloride. Chloride products (**7a**–**7d**) that were highly sensitive to light and moisture were wrapped with aluminium foil, stored in the freezer immediately once prepared, and used in the next step without further purification. Stable chloride products (**7f**–**7j**) were purified by gradient column chromatography.

##### 4.2.5.1 2-(Chloromethyl)-1,4-bis(4-fluorobenzyl)piperazine (**7a**)

Prepared from (1,4-bis(4-fluorobenzyl)piperazin-2-yl)methanol (**6a**) (1.00 g, 3.00 mmol). The product was obtained as a brown solid, yield 0.52 g (50%). M.p. 66–68 °C. TLC (95:5 CH_2_Cl_2_/MeOH), R*f* = 0.91. ^1^H NMR (DMSO-*d*_6_): *δ* 7.36–7.12 (m, 8H, Ar), 3.93 (m, 2H, H-9_a,b_), 3.84 (d, *J* = 13.2 Hz, 1H, H-7_a_), 3.49 (d, *J* = 13.3 Hz, 1H, H-7_b_), 3.41 (m, 2H, H-8_a,b_), 2.71 (m, 1H, H-2), 2.61 (m, 1H, H-3_a_), 2.48 (m, 1H, H-3_b_), 2.31 (m, 4H, H-6_a,b_, H-5_a,b_). ^13^C NMR (DMSO-*d*_6_): *δ* 162.7 (2 × C, Ar), 139.3 (2 × C, Ar), 129.1 (2 × CH, Ar), 128.7 (2 × CH, Ar), 115.5 (2 × CH, Ar), 115.3 (2 × CH, Ar), 61.5 (CH_2_), 59.7 (CH, piperazine), 57.5 (CH_2_), 54.9 (CH_2_, piperazine), 54.7 (CH_2_, piperazine), 49.1 (CH_2_, piperazine), 43.4 (CH_2_). ^19^F NMR (DMSO-*d*_6_): *δ* −115.99 (2 × C_6_H_5_F). [ESI-HRMS] calculated for C_19_H_22_ClF_2_N_2_: 351.1440 [M+H]^+^. Found: 351.1449 [M+H]^+^.

##### 4.2.5.2 2-(Chloromethyl)-1,4-bis(4-chlorobenzyl)piperazine (**7b**)

Prepared from (1,4-bis(4-chlorobenzyl)piperazin-2-yl)methanol (**6b**) (0.3 g, 0.82 mmol). The product was obtained as a brown solid, yield 0.21 g (68%). M.p. 64–66 °C. TLC (95:5 CH_2_Cl_2_/MeOH), R*f* = 0.92. ^1^H NMR (DMSO-*d*_6_): *δ* 7.33 (m, 8H, Ar), 3.95 (m, 2H, H-9_a,b_), 3.48–3.36 (m, 4H, H-7_a,b_, H-8_a,b_), 2.72 (m, 1H, H-2), 2.60 (m, 1H, H-3_a_), 2.49 (m, 1H, H-3_b_), 2.38 (m, 2H, H-6_a,b_), 2.28 (m, 2H, H-5_a,b_). ^13^C NMR (DMSO-*d*_6_): *δ* 139.1 (2 × C, Ar), 131.8 (2 × C, Ar), 130.8 (2 × CH, Ar), 129.1 (2 × CH, Ar), 128.6 (2 × CH, Ar), 127.3 (2 × CH, Ar), 62.4 (CH_2_), 59.7 (CH, piperazine), 58.1 (CH_2_), 57.5 (CH_2_, piperazine), 52.8 (CH_2_, piperazine), 49.2 (CH_2_, piperazine), 43.3 (CH_2_). [ESI-HRMS] calculated for C_19_H_22_Cl_3_N_2_: 383.0849 [M+H]^+^. Found: 383.0856 [M+H]^+^.

##### 4.2.5.3 2-(Chloromethyl)-1,4-bis(4-bromobenzyl)piperazine (**7c**)

Prepared from (1,4-bis(4-bromobenzyl)piperazin-2-yl)methanol (**6c**) (0.80 g, 1.76 mmol). The product was obtained as a light yellow solid, yield 0.53 g (64%). M.p. 70–72 °C. TLC (95:5 CH_2_Cl_2_/MeOH), R*f* = 0.94. ^1^H NMR (DMSO-*d*_6_): *δ* 7.50 (d, *J* = 8.3 Hz, 4H, Ar), 7.27 (d, *J* = 8.3 Hz, 4H, Ar), 3.98 (m, 2H, H-9_a,b_), 3.85 (m, 2H, H-7_a,b_), 3.45 (m, 2H, H-8_a,b_), 3.40 (m, 1H, H-2), 2.70 (m, 1H, H-3_a_), 2.60 (m, 1H, H-3_b_), 2.45 (m, 1H, H-6_a_), 2.37 (m, 1H, H-6_b_), 2.26 (m, 2H, H-5_a,b_). ^13^C NMR (DMSO-*d*_6_): *δ* 137.7 (2 × C, Ar), 137.2 (2 × C, Ar), 131.4 (2 × CH, Ar), 131.3 (2 × CH, Ar), 130.6 (2 × CH, Ar), 130.4 (2 × CH, Ar), 62.1 (CH_2_), 60.3 (CH, piperazine), 57.5 (CH_2_), 55.1 (CH_2_, piperazine), 52.7 (CH_2_, piperazine), 51.0 (CH_2_, piperazine), 42.3 (CH_2_). [ESI-HRMS] calculated for C_19_H_22_Br_2_ClN_2_: 470.9838 [M+H]^+^. Found: 470.9844 [M+H]^+^.

##### 4.2.5.4 2-(Chloromethyl)-1,4-bis(3,5-dichlorobenzyl)piperazine (**7d**)

Prepared from (1,4-bis(3,5-dichlorobenzyl)piperazin-2-yl)methanol (**6d**) (0.50 g, 1.15 mmol). The product was obtained as a brown oil, yield 0.31 g (60%). TLC (95:5 CH_2_Cl_2_/MeOH), R*f* = 0.99. ^1^H NMR (DMSO-*d*_6_): *δ* 7.33 (m, 6H, Ar), 3.94 (m, 2H, H-9_a,b_), 3.85 (m, 2H, H-7_a,b_), 3.52 (m, 2H, H-8_a,b_), 3.45 (m, 1H, H-2), 2.73 (m, 1H, H-3_a_), 2.63 (m, 1H, H-3_b_), 2.40 (m, 1H, H-6_a_), 2.29 (m, 3H, H-6_b_, H-5_a,b_). ^13^C NMR (DMSO-*d*_6_): *δ* 142.1 (2 × C, Ar), 141.3 (2 × C, Ar), 133.4 (2 × C, Ar), 130.5 (2 × CH, Ar), 128.7 (CH, Ar), 128.6 (CH, Ar), 127.7 (CH, Ar), 127.3 (CH, Ar), 61.5 (CH_2_), 59.7 (CH, piperazine), 57.6 (CH_2_), 56.7 (CH_2_, piperazine), 54.9 (CH_2_, piperazine), 52.8 (CH_2_, piperazine), 43.4 (CH_2_). [ESI-HRMS] calculated for C_19_H_20_Cl_5_N_2_: 451.0069 [M+H]^+^. Found: 451.0072 [M+H]^+^.

##### 4.2.5.5 2-(Chloromethyl)-1,4-bis(4-ethylbenzyl)piperazine (**7f**)

Prepared from (1,4-bis(4-ethylbenzyl)piperazin-2-yl)methanol (**6f**) (1.35 g, 3.82 mmol). The product was purified by gradient column chromatography, eluting with petroleum ether –EtOAc 4:1 v/v to give the product as a brown solid, yield 1.05 g (74%). M.p. 80–82 °C. TLC (3:1 petroleum ether/EtOAc), R*f* = 0.61. ^1^H NMR (DMSO-*d*_6_): *δ* 7.18 (m, 8H, Ar), 3.95 (m, 2H, H-9_a,b_), 3.85 (m, 2H, H-7_a,b_), 3.42 (m, 2H, H-8_a,b_), 2.67 (m, 1H, H-2), 2.61 (m, 1H, H-3_a_), 2.57 (q, *J* = 7.1 Hz, 4H, 2 × CH_2_CH_3_), 2.44 (m, 1H, H-3_b_), 2.36 (m, 1H, H-6_a_), 2.25 (m, 3H, H-6_b_, H-5_a,b_), 1.16 (t, *J* = 7.7 Hz, 6H, 2 × CH_3_). ^13^C NMR (125 MHz, DMSO-*d*_6_): *δ* 142.7 (2 × C, Ar), 136.2 (2 × C, Ar), 129.1 (4 × CH, Ar), 128.0 (4 × CH, Ar), 62.2 (CH_2_), 59.7 (CH, piperazine), 57.2 (CH_2_), 57.1 (CH_2_, piperazine), 52.8 (CH_2_, piperazine), 49.3 (CH_2_, piperazine), 43.5 (CH_2_), 28.29 (2 × CH_2_CH_3_), 16.08 (2 × CH_2_CH_3_). [ESI-HRMS] calculated for C_23_H_32_ClN_2_: 371.2249 [M+H]^+^. Found: 371.2261 [M+H]^+^.

##### 4.2.5.6 2-(Chloromethyl)-1,4-bis(4-isopropylbenzyl)piperazine (**7g**)

Prepared from (1,4-bis(4-isopropylbenzyl)piperazin-2-yl)methanol (**6g**) (1.50 g, 3.94 mmol). The product was purified by gradient column chromatography, eluting with petroleum ether –EtOAc 4:1 v/v to give the product as a brown solid, yield 1.37 g (88%). M.p. 64–66 °C. TLC (3:1 petroleum ether/EtOAc), R*f* = 0.64. ^1^H NMR (DMSO-*d*_6_): *δ* 7.19 (m, 8H, Ar), 3.95 (m, 2H, H-9_a,b_), 3.85 (m, 2H, H-7_a,b_), 3.41 (m, 2H, H-8_a,b_), 2.85 (m, 2H, 2 × CH(CH_3_)_2_), 2.68 (m, 1H, H-2), 2.60 (m, 1H, H-3_a_), 2.44 (m, 1H, H-3_b_), 2.36 (m, 1H, H-6_a_), 2.24 (m, 3H, H-6_b_, H-5_a,b_), 1.19 (s, 6H, 2 × CH_3_), 1.18 (s, 6H, 2 × CH_3_). ^13^C NMR (DMSO-*d*_6_): *δ* 147.5 (2 × C, Ar), 136.3 (2 × C, Ar), 129.1 (4 × CH, Ar), 126.5 (4 × CH, Ar), 62.2 (CH_2_), 59.7 (CH, piperazine), 57.19 (CH_2_), 55.4 (CH_2_, piperazine), 52.8 (CH_2_, piperazine), 49.2 (CH_2_, piperazine), 43.4 (CH_2_), 33.6 (2 × CH(CH_3_)_2_), 24.4 (4 × CH_3_). [ESI-HRMS] calculated for C_25_H_36_ClN_2_: 399.2562 [M+H]^+^. Found: 399.2569 [M+H]^+^.

##### 4.2.5.7 2-(Chloromethyl)-1,4-bis(4-(*tert*-butyl)benzyl)piperazine (**7h**)

Prepared from (1,4-bis(4-*tert*butylbenzyl)piperazin-2-yl)methanol (**6h**) (2.0 g, 4.89 mmol). The product was purified by gradient column chromatography, eluting with petroleum ether –EtOAc 4:1 v/v to give the product as a brown solid, yield 1.72 g (83%). M.p. 110–112 °C. TLC (3:1 petroleum ether/EtOAc), R*f* = 0.66. ^1^H NMR (DMSO-*d*_6_): *δ* 7.25 (m, 8H, Ar), 3.97 (m, 2H, H-9_a,b_), 3.43 (d, *J* = 13.2 Hz, 1H, H-7_a_), 3.31 (d, *J* = 13.1 Hz, 1H, H-7_b_), 2.79 (d, *J* = 12.6 Hz, 1H, H-8_a_), 2.61 (d, *J* = 12.7 Hz, 1H, H-8_b_), 2.46 (m, 3H, H-2, H-3_a,b_), 2.22 (m, 4H, H-6_a,b_, H-5_a,b_), 1.28 (s, 9H, 3 × CH_3_), 1.27 (s, 9H, 3 × CH_3_). ^13^C NMR (DMSO-*d*_6_): *δ* 149.3 (2 × C, Ar), 136.0 (2 × C, Ar), 128.9 (4 × CH, Ar), 125.1 (4 × CH, Ar), 61.9 (CH_2_), 61.7 (CH, piperazine), 57.8 (CH_2_), 56.6 (CH_2_, piperazine), 53.2 (CH_2_, piperazine), 50.7 (CH_2_, piperazine), 43.5 (CH_2_), 34.5 (2 × C(CH_3_)_3_), 31.5 (6 × CH_3_). [ESI-HRMS] calculated for C_27_H_40_ClN_2_: 427.2875 [M+H]^+^. Found: 427.2889 [M+H]^+^.

##### 4.2.5.8 2-(Chloromethyl)-1,4-bis(pyridine-4-ylmethyl)piperazine (**7i**)

Prepared from (1,4-bis(pyridine-4-yl)piperazin-2-yl)methanol (**6i**) (0.60 g, 2.01 mmol). The product was purified by gradient column chromatography eluting with petroleum ether – EtOAc 4:1 v/v to give the product as a colourless oil, yield 0.42 g (66%). TLC (2:1 petroleum ether/EtOAc), R*f* = 0.59. ^1^H NMR (DMSO-*d*_6_): *δ* 8.50 (m, 4H, pyridine), 7.34 (m, 4H, pyridine), 3.98 (m, 2H, H-9_a,b_), 3.85 (d, *J* = 13.9 Hz, 1H, H-7_a_), 3.55 (d, *J* = 13.5 Hz, 1H, H-7_b_), 3.50 (m, 2H, H-8_a,b_), 2.77 (m, 1H, H-2), 2.65 (m, 1H, H-3_a_), 2.56 (m, 1H, H-3_b_), 2.43 (m, 1H, H-6_a_), 2.30 (m, 3H, H-6_b_, H-5_a,b_). ^13^C NMR (DMSO-*d*_6_): *δ* 150.0 (2 × CH, pyridine), 149.9 (2 × CH, pyridine), 148.6 (2 × C, pyridine), 124.2 (2 × CH, pyridine), 124.0 (2 × CH, pyridine), 60.9 (CH_2_), 59.7 (CH, piperazine), 56.3 (CH_2_), 55.4 (CH_2_, piperazine), 52.8 (CH_2_, piperazine), 49.2 (CH_2_, piperazine), 43.3 (CH_2_). [ESI-HRMS] calculated for C_17_H_22_ClN_4_: 317.1533 [M+H]^+^. Found: 317.1546 [M+H]^+^.

##### 4.2.5.9 2-(Chloromethyl)-1,4-bis(pyridine-3-ylmethyl)piperazine (**7j**)

Prepared from (1,4-bis(pyridine-3-yl)piperazin-2-yl)methanol (**6j**) (0.80 g, 2.68 mmol). The product was purified by gradient column chromatography eluting with petroleum ether – EtOAc 4:1 v/v to give the product as a colourless oil, yield 0.68 g (81%). TLC (2:1 petroleum ether/EtOAc), R*f* = 0.58. ^1^H NMR (DMSO-*d*_6_): *δ* 8.48 (m, 4H, pyridine), 7.71 (t, *J* = 5.4 Hz, 2H, pyridine), 7.35 (d, *J* = 5.5 Hz, 2H, pyridine), 3.98 (m, 2H, H-9_a,b_), 3.87 (d, *J* = 13.4 Hz, 1H, H-7_a_), 3.54 (d, *J* = 13.5 Hz, 1H, H-7_b_), 3.47 (m, 2H, H-8_a,b_), 2.72 (m, 1H, H-2), 2.62 (m, 1H, H-3_a_), 2.40 (m, 1H, H-3_b_), 2.30 (m, 2H, H-6_a,b_), 2.13 (m, 2H, H-5_a,b_). ^13^C NMR (DMSO-*d*_6_): *δ* 150.4 (CH, pyridine), 150.3 (CH, pyridine), 148.8 (CH, pyridine), 148.7 (CH, pyridine), 136.9 (CH, pyridine), 136.8 (CH, pyridine), 134.6 (2 × C, pyridine), 123.9 (2 × CH, pyridine), 62.2 (CH_2_), 60.3 (CH, piperazine), 55.5 (CH_2_), 54.8 (CH_2_, piperazine), 51.3 (CH_2_, piperazine), 48.9 (CH_2_, piperazine), 43.2 (CH_2_). [ESI-HRMS] calculated for C_17_H_22_ClN_4_: 317.1533 [M+H]^+^. Found: 317.1540 [M+H]^+^.

#### 4.2.6 General method for the preparation of imidazole derivatives (8)

To a stirred suspension of K_2_CO_3_ (4 m.eq.) in dry acetonitrile (10 mL/4 mmol of K_2_CO_3_) was added imidazole (4 m.eq.). The reaction mixture was refluxed at 45 °C for 1 h. After cooling to room temperature the chloride (**7**) (1 m.eq.) was added and the reaction mixture refluxed at 70 °C for 48 h. The solvent was evaporated under reduced pressure and the resulting mixture diluted with EtOAc (50 mL/mmol of chloride (**7**) used) and washed with brine (3 × 20 mL/mmol) and H_2_O (3 × 20 mL/mmol). The organic layer was dried (MgSO_4_) and evaporated under reduced pressure to give the crude imidazole, which was purified by gradient column chromatography.

##### 4.2.6.1 2-((1H-Imidazol-1-yl)methyl)-1,4-bis(4-fluorobenzyl)piperazine (**8a**)

Prepared from 2-(chloromethyl)-1,4-bis(4-fluorobenzyl)piperazine (**7a**) (0.50 g, 1.42 mmol). The product was purified by gradient column chromatography eluting with CH_2_Cl_2_-MeOH 98:2 v/v, followed by further purification by preparative TLC (CH_2_Cl_2_-MeOH 95:5 to 9:1 v/v) to give the product as a yellow oil*, yield 0.29 g (53%). TLC (95:5 CH_2_Cl_2_/MeOH), R*f* = 0.50. ^1^H NMR (DMSO-*d*_6_): *δ* 7.47 (s, 1H, imidazole), 7.36–7.11 (m, 8H, Ar), 6.97 (s, 1H, imidazole), 6.82 (s, 1H, imidazole), 4.30 (dd, *J* = 4.1, 13.6 Hz, 1H, H-9_a_), 4.22 (dd, *J* = 7.0, 13.6 Hz, 1H, H-9_b_), 3.93 (d, *J* = 13.6 Hz, 1H, H-7_a_), 3.59 (d, *J* = 13.2 Hz, 1H, H-7_b_), 3.44 (d, *J* = 13.2 Hz, 1H, H-8_a_), 3.32 (d, *J* = 13.2 Hz, 1H, H-8_b_), 2.82 (m, 1H, H-2), 2.74 (m, 1H, H-3_a_), 2.30 (m, 4H, H-3_b_, H-6_a,b_, H-5_a_), 2.12 (m, 1H, H-5_b_). ^13^C NMR (DMSO-*d*_6_): *δ* 139.3 (2 × C, Ar), 134.7 (2 × C, Ar), 131.2 (2 × CH, Ar), 131.1 (2 × CH, Ar), 130.7 (CH, imidazole), 128.9 (2 × CH, Ar), 128.7 (2 × CH, Ar), 127.3 (CH, imidazole), 120.3 (CH, imidazole), 61.6 (CH_2_), 59.3 (CH, piperazine), 57.7 (CH_2_), 56.8 (CH_2_), 55.4 (CH_2_, piperazine), 52.3 (CH_2_, piperazine), 44.3 (CH_2_, piperazine). ^19^F NMR (DMSO-*d*_6_): *δ* −115.87 (2 × C_6_H_4_F). [ESI-HRMS] calculated for C_22_H_25_F_2_N_4_: 383.2047 [M+H]^+^. Found: 383.2050 [M+H]^+^.

* The product was found to be unstable (light/moisture) so was wrapped in aluminium foil and stored in the freezer.

##### 4.2.6.2 2-((1H-Imidazol-1-yl)methyl)-1,4-bis(4-chlorobenzyl)piperazine (**8b**)

Prepared from 2-(chloromethyl)-1,4-bis(4-chlorobenzyl)piperazine (**7b**) (0.15 g, 0.39 mmol). The product was purified by gradient column chromatography eluting with CH_2_Cl_2_-MeOH 97.5:2.5 v/v, followed by further purification by preparative TLC (CH_2_Cl_2_-MeOH 95:5 v/v) to give the product as a colourless oil, yield 0.09 g (56%). TLC (95:5 CH_2_Cl_2_/MeOH), R*f* = 0.60. ^1^H NMR (CDCl_3_): *δ* 7.24 (s, 1H, imidazole), 7.22–7.12 (m, 8H, Ar), 6.89 (s, 1H, imidazole), 6.59 (s, 1H, imidazole), 4.28 (dd, *J* = 4.3, 13.6 Hz, 1H, H-9_a_), 4.22 (dd, *J* = 7.7, 13.6 Hz, 1H, H-9_b_), 3.78 (d, *J* = 13.0 Hz, 1H, H-7_a_), 3.54 (d, *J* = 13.0 Hz, 1H, H-7_b_), 3.36 (d, *J* = 13.1 Hz, 1H, H-8_a_), 3.27 (d, *J* = 12.9 Hz, 1H, H-8_b_), 2.80 (m, 1H, H-2), 2.76 (m, 1H, H-3_a_), 2.42 (m, 1H, H-3_b_), 2.40–2.13 (m, 4H, H-6_a,b_, H-5_a,b_). ^13^C NMR (CDCl_3_): *δ* 138.1 (C, Ar), 136.9 (C, Ar), 136.4 (C, Ar), 133.0 (C, Ar), 130.4 (CH, imidazole), 128.6 (4 × CH, Ar), 128.5 (4 × CH, Ar), 127.3 (CH, imidazole), 120.5 (CH, imidazole), 62.1 (CH_2_), 58.7 (CH, piperazine), 58.3 (CH_2_), 53.8 (CH_2_), 52.2 (CH_2_, piperazine), 48.3 (CH_2_, piperazine), 44.1 (CH_2_, piperazine). [ESI-HRMS] calculated for C_22_H_25_Cl_2_N_4_: 415.1456 [M+H]^+^. Found: 415.1447 [M+H]^+^.

##### 4.2.6.3 2-((1H-Imidazol-1-yl)methyl)-1,4-bis(4-bromobenzyl)piperazine (**8c**)

Prepared from 2-(chloromethyl)-1,4-bis(4-bromobenzyl)piperazine (**7c**) (0.4 g, 0.84 mmol). The product was purified by gradient column chromatography eluting with CH_2_Cl_2_-MeOH 95:5 v/v to give the product as a white solid, yield 0.26 g (62%). M.p. 54–56 °C. TLC (95:5 CH_2_Cl_2_/MeOH), R*f* = 0.68. ^1^H NMR (CDCl_3_): *δ* 7.51 (s, 1H, imidazole), 7.49 (m, 4H, Ar), 7.26 (d, *J* = 8.2 Hz, 2H, Ar), 7.21 (d, *J* = 8.2 Hz, 2H, Ar), 7.00 (s, 1H, imidazole), 6.83 (s, 1H, imidazole), 4.31 (dd, *J* = 4.6, 13.5 Hz, 1H, H-9_a_), 4.20 (dd, *J* = 7.0, 13.5 Hz, 1H, H-9_b_), 3.89 (d, *J* = 14.0 Hz, 1H, H-7_a_), 3.57 (d, *J* = 14.2 Hz, 1H, H-7_b_), 3.43 (d, *J* = 13.4 Hz, 1H, H-8_a_), 3.32 (d, *J* = 13.7 Hz, 1H, H-8_b_), 2.82 (m, 1H, H-2), 2.75 (m, 1H, H-3_a_), 2.30 (m, 4H, H-3_b_, H-6_a,b_, H-5_a_), 2.10 (m, 1H, H-5_b_). ^13^C NMR (CDCl_3_): *δ* 139.0 (2 × C, Ar), 138.0 (2 × C, Ar), 131.5 (CH, imidazole), 131.4 (4 × CH, Ar), 131.0 (4 × CH, Ar), 128.6 (CH, imidazole), 120.4 (CH, imidazole), 61.7 (CH_2_), 59.3 (CH, piperazine), 56.8 (CH_2_), 54.1 (CH_2_), 52.2 (CH_2_, piperazine), 46.5 (CH_2_, piperazine), 44.4 (CH_2_, piperazine). Anal. Calcd for C_22_H_24_Br_2_N_4_ (504.2608): C, 52.40; H, 4.80; N, 11.11. Found: C, 52.30; H, 4.91; N, 11.20.

##### 4.2.6.4 2-((1H-Imidazol-1-yl)methyl)-1,4-bis(3,5-dichlorobenzyl)piperazine (**8d**)

Prepared from 2-(chloromethyl)-1,4-bis(3,5-dichlorobenzyl)piperazine (**7d**) (0.35 g, 0.77 mmol). The product was purified by gradient column chromatography eluting with CH_2_Cl_2_-MeOH 99.8:0.2 v/v followed by further purification by preparative TLC (petroleum ether-EtOAc 3:2 v/v) to give the product as a colourless oil, yield 0.18 g (51%). TLC (1:1 petroleum ether/EtOAc), R*f* = 0.94. ^1^H NMR (CDCl_3_): *δ* 7.47 (s, 1H, imidazole), 7.28 (s, 1H, *para*-Ar), 7.26 (s, 1H, *para*-Ar), 7.21 (s, 2H, Ar), 7.19 (s, 2H, Ar), 7.05 (s, 1H, imidazole), 6.77 (s, 1H, imidazole), 4.29 (dd, *J* = 4.1, 13.6 Hz, 1H, H-9_a_), 4.21 (dd, *J* = 7.0, 13.6 Hz, 1H, H-9_b_), 3.79 (d, *J* = 14.0 Hz, 1H, H-7_a_), 3.64 (d, *J* = 13.9 Hz, 1H, H-7_b_), 3.46 (d, *J* = 13.4 Hz, 1H, H-8_a_), 3.39 (d, *J* = 13.4 Hz, 1H, H-8_b_), 2.96 (m, 1H, H-2), 2.88 (m, 1H, H-3_a_), 2.52 (m, 3H, H-3_b_, H-6_a,b_), 2.35 (m, 2H, H-5_a,b_). ^13^C NMR (CDCl_3_): *δ* 142.1 (2 × C, Ar), 141.5 (2 × C, Ar), 135.1 (2 × C, Ar), 129.3 (CH, imidazole), 127.6 (2 × CH, Ar), 127.2 (2 × CH, Ar), 126.9 (CH, imidazole), 126.6 (2 × CH, Ar), 119.4 (CH, imidazole), 61.8 (CH_2_), 59.0 (CH, piperazine), 57.4 (CH_2_), 53.7 (CH_2_), 52.0 (CH_2_, piperazine), 47.8 (CH_2_, piperazine), 44.2 (CH_2_, piperazine). [ESI-HRMS] calculated for C_22_H_23_Cl_4_N_4_: 483.0677 [M+H]^+^. Found: 483.0678 [M+H]^+^.

##### 4.2.6.5 2-((1H-Imidazol-1-yl)methyl)-1,4-bis(4-methoxybenzyl)piperazine (**8e**)

Prepared from 2-(chloromethyl)-1,4-bis(4-methoxybenzyl)piperazine (**7e**) (0.35 g, 0.93 mmol). The product was purified by gradient column chromatography eluting with CH_2_Cl_2_-MeOH 97.5:2.5 v/v followed by further purification by preparative TLC (CH_2_Cl_2_-MeOH 9:1 v/v) to give the product as a yellow oil, yield 0.25 g (68%). TLC (95:5 CH_2_Cl_2_/MeOH), R*f* = 0.53. ^1^H NMR (DMSO-*d*_6_): *δ* 7.46 (s, 1H, imidazole), 7.19 (d, *J* = 8.5 Hz, 2H, Ar), 7.18 (d, *J* = 8.5 Hz, 2H, Ar), 6.96 (s, 1H, imidazole), 6.87 (d, *J* = 8.2 Hz, 4H, Ar), 6.81 (s, 1H, imidazole), 4.29 (dd, *J* = 4.4, 13.9 Hz, 1H, H-9_a_), 4.20 (dd, *J* = 7.8, 13.9 Hz, 1H, H-9_b_), 3.84 (d, *J* = 13.0 Hz, 1H, H-7_a_), 3.73 (s, 6H, 2 × OCH_3_), 3.51 (d, *J* = 12.9 Hz, 1H, H-7_b_), 3.39 (d, *J* = 12.8 Hz, 1H, H-8_a_), 3.25 (d, *J* = 12.8 Hz, 1H, H-8_b_), 2.77 (m, 1H, H-2), 2.69 (m, 1H, H-3_a_), 2.30–2.22 (m, 4H, H-3_b_, H-6_a,b_, H-5_a_), 2.09 (m, 1H, H-5_b_). ^13^C NMR (DMSO-*d*_6_): *δ* 142.8 (C, Ar), 142.7 (C, Ar), 134.6 (C, Ar), 131.0 (C, Ar), 130.5 (2 × CH, Ar), 130.1 (2 × CH, Ar), 128.6 (CH, imidazole), 128.1 (CH, imidazole), 120.1 (CH, imidazole), 114.1 (2 × CH, Ar), 114.0 (2 × CH, Ar), 62.0 (CH_2_), 59.2 (CH, piperazine), 58.0 (CH_2_), 57.0 (CH_2_), 55.5 (2 × OCH_3_), 54.8 (CH_2_, piperazine), 54.3 (CH_2_, piperazine), 52.3 (CH_2_, piperazine). [ESI-HRMS] calculated for C_24_H_31_N_4_O_2_: 407.2442 [M+H]^+^. Found: 407.2438 [M+H]^+^.

##### 4.2.6.6 2-((1H-imidazol-1-yl)methyl)-1,4-bis(4-ethylbenzyl)piperazine (**8f**)

Prepared from 2-(chloromethyl)-1,4-bis(4-ethylbenzyl)piperazine (**7f**) (1.50 g, 4.04 mmol). The product was purified by gradient column chromatography eluting with CH_2_Cl_2_-MeOH 99.5:0.5 v/v followed by further purification by preparative TLC (CH_2_Cl_2_-MeOH 95:5 v/v) to give the product as a yellow oil, yield 1.12 g (69%). TLC (95:5 CH_2_Cl_2_/MeOH), R*f* = 0.51. ^1^H NMR (DMSO-*d*_6_): *δ* 7.45 (s, 1H, imidazole), 7.16 (m, 8H, Ar), 6.93 (s, 1H, imidazole), 6.80 (s, 1H, imidazole), 4.29 (dd, *J* = 4.3, 13.8 Hz, 1H, H-9_a_), 4.21 (dd, *J* = 7.7, 13.8 Hz, 1H, H-9_b_), 3.88 (d, *J* = 13.3 Hz, 1H, H-7_a_), 3.54 (d, *J* = 13.3 Hz, 1H, H-7_b_), 3.4 (d, *J* = 13.1 Hz, 1H, H-8_a_), 3.27 (d, *J* = 12.9 Hz, 1H, H-8_b_), 2.78 (m, 1H, H-2), 2.71 (m, 1H, H-3_a_), 2.58 (m, 4H, 2 × CH_2_CH_3_), 2.31–2.24 (m, 3H, H-3_b_, H-6_a,b_), 2.11 (m, 2H, H-5_a,b_), 1.17 (t, *J* = 7.58 Hz, 3H, CH_2_CH_3_), 1.16 (t, *J* = 7.6 Hz, 3H, CH_2_CH_3_). ^13^C NMR (DMSO-*d*_6_): *δ* 142.9 (C, Ar), 142.7 (C, Ar), 136.5 (C, Ar), 135.7 (C, Ar), 129.4 (2 × CH, Ar), 128.9 (2 × CH, Ar), 128.5 (CH, imidazole), 128.1 (2 × CH, Ar), 128.0 (2 × CH, Ar), 126.4 (CH, imidazole), 120.3 (CH, imidazole), 62.3 (CH_2_), 59.3 (CH, piperazine), 58.8 (CH_2_), 57.4 (CH_2_), 55.4 (CH_2_, piperazine), 54.3 (CH_2_, piperazine), 52.4 (CH_2_, piperazine), 28.3 (CH_2_CH_3_), 28.2 (CH_2_CH_3_), 16.2 (CH_2_CH_3_), 16.1 (CH_2_CH_3_). [ESI-HRMS] calculated for C_26_H_35_N_4_: 403.2862 [M+H]^+^. Found: 403.2857 [M+H]^+^.

##### 4.2.6.7 2-((1H-Imidazol-1-yl)methyl)-1,4-bis(4-isopropylbenzyl)piperazine (**8g**)

Prepared from 2-(chloromethyl)-1,4-bis(4-isopropylbenzyl)piperazine (**7g**) (1.50 g, 3.75 mmol). The product was purified by gradient column chromatography eluting with CH_2_Cl_2_-MeOH 99.7:0.3 v/v followed by further purification by preparative TLC (petroleum ether-EtOAc 3:2 v/v) to give the product as a brown solid, yield 1.02 g (63%). M.p. 110–112 °C. TLC (2:1 petroleum ether/EtOAc), R*f* = 0.53. ^1^H NMR (DMSO-*d*_6_): *δ* 7.43 (s, 1H, imidazole), 7.19 (m, 8H, Ar), 6.90 (s, 1H, imidazole), 6.79 (s, 1H, imidazole), 4.28 (dd, *J* = 4.4, 14.0 Hz, 1H, H-9_a_), 4.21 (dd, *J* = 7.8, 14.0 Hz, 1H, H-9_b_), 3.87 (d, *J* = 13.3 Hz, 1H, H-7_a_), 3.55 (d, *J* = 13.3 Hz, 1H, H-7_b_), 3.43 (d, *J* = 13.0 Hz, 1H, H-8_a_), 3.26 (d, *J* = 13.0 Hz, 1H, H-8_b_), 2.85 (m, 2H, 2 × CH(CH_3_)_2_), 2.78 (m, 1H, H-2), 2.71 (m, 1H, H-3_a_), 2.32–2.24 (m, 3H, H-3_b_, H-6_a,b_), 2.10 (m, 2H, H-5_a,b_), 1.98 (d, *J* = 6.9 Hz, 6H, 2 × CH_3_), 1.89 (d, *J* = 6.8 Hz, 6H, 2 × CH_3_). ^13^C NMR (DMSO-*d*_6_): *δ* 147.5 (C, Ar), 147.4 (C, Ar), 136.6 (C, Ar), 135.9 (C, Ar), 129.4 (2 × CH, Ar), 128.9 (2 × CH, Ar), 128.5 (CH, imidazole), 126.6 (2 × CH, Ar), 126.5 (2 × CH, Ar), 126.4 (CH, imidazole), 120.3 (CH, imidazole), 62.3 (CH_2_), 59.4 (CH, piperazine), 57.4 (CH_2_), 56.9 (CH_2_), 55.4 (CH_2_, piperazine), 54.3 (CH_2_, piperazine), 52.5 (CH_2_, piperazine), 33.6 (2 × CH(CH_3_)_2_), 24.4 (4 × CH_3_). [ESI-HRMS] calculated for C_28_H_39_N_4_: 431.3175 [M+H]^+^. Found: 431.3186 [M+H]^+^.

##### 4.2.6.8 2-((1H-Imidazol-1-yl)methyl)-1,4-bis(4-(*tert*-butyl)benzyl)piperazine (**8h**)

Prepared from 2-(chloromethyl)-1,4-bis(4-*tert*butylbenzyl)piperazine (**7h**) (1.50 g, 3.51 mmol). The product was purified by gradient column chromatography eluting with CH_2_Cl_2_-MeOH 99.8:0.2 v/v followed by further purification by preparative TLC (petroleum ether-EtOAc 3:2 v/v) to give the product as a yellow oil, yield 0.85 g (53%). TLC (2:1 petroleum ether/EtOAc), R*f* = 0.55. ^1^H NMR (DMSO-*d*_6_): *δ* 7.43 (s, 1H, imidazole), 7.33 (m, 4H, Ar), 7.20 (m, 4H, Ar), 6.89 (s, 1H, imidazole), 6.78 (s, 1H, imidazole), 4.28 (dd, *J* = 4.3, 13.8 Hz, 1H, H-9_a_), 4.21 (dd, *J* = 7.6, 13.8 Hz, 1H, H-9_b_), 3.87 (d, *J* = 13.7 Hz, 1H, H-7_a_), 3.56 (d, *J* = 13.7 Hz, 1H, H-7_b_), 3.44 (d, *J* = 12.8 Hz, 1H, H-8_a_), 3.27 (d, *J* = 12.8 Hz, 1H, H-8_b_), 2.80 (m, 1H, H-2), 2.72 (m, 1H, H-3_a_), 2.36 (m, 1H, H-3_b_), 2.29 (m, 2H, H-6_a_,_b_), 2.11 (m, 2H, H-5_a,b_), 1.28 (s, 9H, 3 × CH_3_), 1.27 (s, 9H, 3 × CH_3_). ^13^C NMR (DMSO-*d*_6_): *δ* 149.8 (C, Ar), 149.6 (C, Ar), 136.2 (C, Ar), 135.5 (C, Ar), 129.1 (2 × CH, Ar), 128.6 (2 × CH, Ar), 128.5 (CH, imidazole), 125.4 (2 × CH, Ar), 125.3 (2 × CH, Ar), 125.2 (CH, imidazole), 120.3 (CH, imidazole), 62.2 (CH_2_), 59.4 (CH, piperazine), 57.4 (CH_2_), 56.3 (CH_2_), 55.4 (CH_2_, piperazine), 54.3 (CH_2_, piperazine), 52.5 (CH_2_, piperazine), 34.6 (2 × C(CH_3_)_3_), 31.7 (6 × CH_3_). [ESI-HRMS] calculated for C_30_H_43_N_4_: 459.3488 [M+H]^+^. Found: 459.3490 [M+H]^+^.

##### 4.2.6.9 2-((1H-Imidazol-1-yl)methyl)-1,4-bis(pyridine-4-ylmethyl)piperazine (**8i**)

Prepared from 2-(chloromethyl)-1,4-bis(pyridine-4-ylmethyl)piperazine (**7i**) (0.4 g, 1.26 mmol). The product was purified by gradient column chromatography, eluting with CH_2_Cl_2_-MeOH-Et_3_N 94:5:1 v/v/v, followed by further purification by preparative TLC (CH_2_Cl_2_-MeOH 9:1 to 85:15 v/v) to give the product as a yellow oil, yield 0.26 g (58%). TLC (1:9 CH_2_Cl_2_/MeOH), R*f* = 0.57. ^1^H NMR (DMSO-*d*_6_): *δ* 8.47 (m, 4H, pyridine), 7.33 (s, 1H, imidazole), 7.15 (m, 4H, pyridine), 6.94 (s, 1H, imidazole), 6.68 (s, 1H, imidazole), 4.28 (dd, *J* = 4.4, 14.0 Hz, 1H, H-9_a_), 4.21 (dd, *J* = 7.8, 14.0 Hz, 1H, H-9_b_), 3.79 (d, *J* = 14.0 Hz, 1H, H-7_a_), 3.58 (d, *J* = 14.0 Hz, 1H, H-7_b_), 3.49 (d, *J* = 13.4 Hz, 1H, H-8_a_), 3.40 (d, *J* = 13.4 Hz, 1H, H-8_b_), 2.88 (m, 1H, H-2), 2.83 (m, 1H, H-3_a_), 2.45–2.21 (m, 5H, H-3_b_, H-6_a,b_, H-5_a,b_). ^13^C NMR (CDCl_3_): *δ* 150.0 (2 × CH, pyridine), 149.9 (2 × CH, pyridine), 137.5 (CH, imidazole), 137.3 (C, pyridine), 137.0 (C, pyridine), 129.5 (CH, imidazole), 123.8 (2 × CH, pyridine), 123.2 (2 × CH, pyridine), 119.3 (CH, imidazole), 61.7 (CH_2_), 59.2 (CH, piperazine), 57.3 (CH_2_), 54.0 (CH_2_), 52.0 (CH_2_, piperazine), 48.1 (CH_2_, piperazine), 44.5 (CH_2_, piperazine). [ESI-HRMS] calculated for C_20_H_25_N_6_: 349.2141 [M+H]^+^. Found: 349.2146 [M+H]^+^.

##### 4.2.6.10 2-((1H-Imidazol-1-yl)methyl)-1,4-bis(pyridine-3-ylmethyl)piperazine (**8j**)

Prepared from 2-(chloromethyl)-1,4-bis(pyridine-3-ylmethyl)piperazine (**7j**) (0.44 g, 1.41 mmol). The product was purified by gradient column chromatography, eluting with CH_2_Cl_2_-MeOH-Et_3_N 94:5:1 v/v/v, followed by further purification by preparative TLC (CH_2_Cl_2_-MeOH 9:1 to 85:15 v/v) to give the product as a yellow oil, yield 0.25 g (53%). TLC (1:9 CH_2_Cl_2_/MeOH), R*f* = 0.56. ^1^H NMR (DMSO-*d*_6_): *δ* 8.50 (s, 1H, pyridine), 8.49 (s, 1H, pyridine), 8.46 (m, 2H, pyridine), 7.71 (m, 2H, pyridine), 7.61 (s, 1H, imidazole), 7.50 (s, 1H, imidazole), 7.34 (m, 2H, pyridine), 6.82 (s, 1H, imidazole), 4.29 (dd, *J* = 4.4, 13.8 Hz, 1H, H-9_a_), 4.21 (dd, *J* = 7.7, 13.8 Hz, 1H, H-9_b_), 3.94 (d, *J* = 13.8 Hz, 1H, H-7_a_), 3.64 (d, *J* = 13.8 Hz, 1H, H-7_b_), 3.49 (d, *J* = 13.4 Hz, 1H, H-8_a_), 3.39 (d, *J* = 13.4 Hz, 1H, H-8_b_), 2.86 (m, 1H, H-2), 2.75 (m, 2H, H-3_a,b_), 2.32 (m, 3H, H-6_a,b_,H-5_a_), 2.13 (m, 1H, H-5_b_). ^13^C NMR (DMSO-*d*_6_): *δ* 150.2 (CH, pyridine), 149.6 (CH, pyridine), 148.6 (CH, pyridine), 148.5 (CH, pyridine), 137.3 (C, pyridine), 137.0 (C, pyridine), 136.4 (CH, imidazole), 135.3 (CH, pyridine), 129.0 (CH, pyridine), 124.0 (CH, imidazole), 121.8 (2 × CH, pyridine), 119.4 (CH, imidazole), 60.1 (CH_2_), 58.8 (CH, piperazine), 55.5 (CH_2_), 53.3 (CH_2_), 51.9 (CH_2_, piperazine), 47.6 (CH_2_, piperazine), 44.1 (CH_2_, piperazine). [ESI-HRMS] calculated for C_20_H_25_N_6_: 349.2141 [M+H]^+^. Found: 349.2149 [M+H]^+^.

### 4.3 CYP121A1 spectral binding assays for ligand K_D_ determination

CYP121A1 protein was expressed and purified as described previously.^25^ Ligand binding assays were performed by spectrophotometric titration using a Cary 60 UV–visible scanning spectrophotometer (Agilent, UK) and a 1 cm path length quartz cuvette, recording spectra between 250 and 800 nm. Titrations were typically done with 3–5 μM CYP121A1 at 25 °C in 100 mM potassium phosphate (KPi) buffer, 200 mM KCl, pH 7.85 with 0.004% Triton X-100. Ligand stock solutions were prepared in dimethylsulfoxide (DMSO). Ligands were added in small volumes (typically 0.05–0.2 µL aliquots) from concentrated stock solutions to the protein in a 1 mL final volume. Spectral measurements were taken before ligand addition, and following addition of each aliquot of ligand until no further spectral change occurred. Difference spectra at each stage in the titration were obtained by subtraction of the initial ligand-free enzyme spectrum from subsequent spectra collected after each addition of ligand. From the difference spectra, a pair of wavelengths were identified and defined as the absorbance maximum (A_peak_) and minimum (A_trough_). The overall absorbance change (ΔA_max_) was calculated by subtracting the A_trough_ value from the A_peak_ value for each spectrum collected after a ligand addition. Graphs of ΔAmax against [ligand] were plotted for each titration. Titrations were done in triplicate and the final *K*_D_ value presented was determined as the average value across the three sets. The *K*_D_ values were determined by fitting the data using either a standard hyberbolic function (Eq. (1)) or the Hill equation (Eq. (2)) using Origin software (OriginLab, Northampton, MA).

(1) $${\text{A}}_{\text{obs}}=\;\left({\text{A}}_{\text{max}}*\text{L}/\left({\text{K}}_{\text{d}}+\text{L}\right)\right)$$

In Eq. (1) (the standard hyperbolic function, the Michaelis-Menten function adapted for ligand binding), A_obs_ is the observed absorbance change at ligand concentration L, A_max_ is the maximal absorbance change observed at apparent ligand saturation, and *K*_d_ is the dissociation constant for the binding of the ligand (the substrate concentration at which A_obs_ = 0.5 × A_max_).

(2) $${\text{A}}_{\text{obs}}=\left({\text{A}}_{\text{max}}\times {\text{L}}^{\text{n}}\right)/\left({\text{K}}^{\text{n}}+{\text{L}}^{\text{n}}\right)$$

In Eq. (2) (the sigmoidal Hill equation), A_obs_ is the observed absorbance change at ligand concentration L, A_max_ is the absorbance change at apparent ligand saturation, *K* is the apparent dissociation constant, and n is the Hill coefficient, a value describing the apparent extent of cooperativity observed in ligand binding.

*Antimycobacterial activity assay*: *M. tuberculosis* H_37_Rv was grown in 7H9 liquid medium with 10% Middlebrook OADC Growth Supplement enrichment (BBL/Becton-Dickinson, Sparks, MD, USA). Cells were cultured at 37 °C until mid-log phase was reached (OD_600nm_ = 0.4–0.6). After cells reached mid-log phase, bacterial suspensions were prepared as described below and REMA assays were performed. The anti-*M. tuberculosis* activities of the compounds were determined by the REMA (Resazurin Microtiter Assay) method.^26^ Stock solutions of the tested compounds (10 mg/mL) were prepared in DMSO and diluted in Middlebrook 7H9 broth supplemented with 10% OADC. The microdilution of the compounds was performed in 96-well plates to obtain final compound concentration ranges of 0.39–100 µg/mL. Rifampicin in the concentration range between 0.004 and 1 µg/mL was added as control. Bacterial suspensions were prepared and their turbidity adjusted to match the optical density of McFarland no. 1 standard. After a further dilution of 1:20 in Middlebrook 7H9 broth supplemented with OADC, 100 µL of the inoculum were added to each well of the 96-well plate. Cultures were incubated for 7 days at 37 °C, and 30 µL of 0.01% resazurin was added. Wells were read after 24 h for colour change and measured as the fluorescence (excitation/emission of 530/590 nm filters, respectively) in a microfluorimeter. The MIC was defined as the lowest concentration resulting in 90% inhibition of *M. tuberculosis* growth. The presented results are representative from two independent experiments.

### 4.4 Computational methods

#### 4.4.1 Molecular modeling and Docking

Docking studies were performed using the MOE^22^ program and Mtb CYP121A1 co-crystallised with cYY (PDB 3G5H). All minimisations were performed with MOE until a RMSD gradient of 0.01 Kcal/mol/A with the MMFF94 forcefield and partial charges were automatically calculated. The charge of the haem iron at physiological pH was set to 3^+^ (geometry d2sp3) through the atom manager in MOE. The Alpha Triangle placement, which derives poses by random superposition of ligand atom triplets through alpha sphere dummies in the receptor site, was chosen to determine the poses. The London ΔG scoring function estimates the free energy of binding of the ligand from a given pose. Refinement of the results was done using the MMFF94 forcefield, and rescoring of the refined results using the London ΔG scoring function was applied. The output database dock file was created with different poses for each ligand and arranged according to the final score function (S), which is the score of the last stage that was not set to zero.

#### 4.4.2 Molecular dynamics simulation

Molecular dynamics simulations were run on the CYP121A1 protein in complex with **8g** and **8h**. Both PDB files were first optimised with protein preparation wizard in Maestro,^27^ version 11.8.012 by assigning bond orders, adding hydrogen, and correcting incorrect bond types. A default quick relaxation protocol was used to minimise the MD systems with the Desmond programme.^28^ In Desmond, the volume of space in which the simulation takes place, the global cell, is built up by regular 3D simulation boxes, which was utilised as part in this system for protein interactions. The orthorhombic water box allowed for a 10 Å buffer region between protein atoms and box sides. Overlapping water molecules were deleted, and the systems were neutralised with Na^+^ ions and salt concentration 0.15 M. Force-field parameters for the complexes were assigned using the OPLS_2005 forcefield, that is, a 50 ns molecular dynamic run in the NPT ensemble (T = 300 K) at a constant pressure of 1 bar. Energy and trajectory atomic coordinate data were recorded at each 1.2 ns.
