# Supplementary material for: Synthesis and biological evaluation of novel cYY analogues targeting Mycobacterium tuberculosis CYP121A1
Source: Bioorg Med Chem. 2019 Apr 15;27(8):1546–61. doi: 10.1016/j.bmc.2019.02.051 (PMC7049898; doi:10.1016/j.bmc.2019.02.051)
Supplement: Supplementary data 1 [file mmc1.docx]

**Synthesis and Biological Evaluation of Novel cYY Analogues targeting *Mycobacterium tuberculosis* CYP121A1**

SUPPORTING INFORMATION

Safaa M. Kishk, Kirsty J. McLean, Sakshi Sood, Mohamed A. Helal, Mohamed S. Gomaa, Ismail Salama, Samia M. Mostafa, Luiz Pedro S. de Carvalho, Andrew W. Munro and Claire Simons

| Synthetic procedures for compounds **3a**-**3c**, **3e**, **3g**-**3j**, **4a**-**4c**, **4e**-**4f**, **4i**, **5e**, **6e** and **7e** | S2 |
| --- | --- |
| References | S12 |
| **Figure S1**. 2-((1*H*-Imidazol-1-yl)methyl)-1,4-bis(4-(tert-butyl)benzyl)piperazine (**8h**) 1D (^1^H) and 2D (COSY) NMR spectra illustrating the coupling interactions between protons | S14 |

**4.1 General Experimental**

All chemicals, reagents and solvents were purchased from Sigma-Aldrich, Fisher Scientific, Alfa-Aesar, Fluka and Acros Chemicals. Whenever required, solvents were dried prior to use as described by the handbook Purification of Laboratory Chemicals and stored over 4 Å molecular sieves under nitrogen. Flash column chromatography was performed with silica gel (230-400 mesh) (Merck) and TLC was performed on pre-coated silica gel plates (Merck Kiesel gel 60_F254_, BDH). Melting points were determined on an electrothermal instrument (Gallenkamp), and are uncorrected. Compounds were visualised by irradiation with UV light at 254 nm and 365 nm or by using KMnO_4_ stain or vanillin stain followed by heating. NMR spectra were recorded on a Bruker AVANCE DPX500 spectrometer operating at 500 and 125 MHz for ^1^H and ^13^C NMR, respectively, and auto calibrated to the deuterated solvent reference peak. The assignments were made using one-dimensional (1D) and two-dimensional (2D) HSQC and COSY spectra. Chemical shifts are given in δ relative to tetramethylsilane (TMS); the coupling constants (*J*) are given in Hertz. TMS was used as an internal standard (δ = 0 ppm) for ^1^H NMR and CDCl_3_ served as an internal standard (δ = 77.0 ppm) for ^13^C NMR. Multiplicity is denoted as s (singlet), br s (broad singlet), d (doublet), dd (doublet of doublet), t (triplet), q (quartet), m (multiplet) or combinations thereof. All compounds were more than 95% pure.

***General method for the preparation of diimines (3)***. To a stirred solution of benzaldehyde (**1**) (2 m.eq.) in ethanol (1mL/mmol of benzaldehyde), was added ethylenediamine (**2**) (1 m.eq.) dropwise. The reaction mixture was stirred for 6 h at room temperature, and the precipitated white coloured crystals were collected by filtration, washed with diethyl ether and dried to give the desired diimine.

*N,N´-(Ethane-1,2-diyl)bis(1-(4-fluorophenyl)methanimine) (****3a****)*

(C_16_H_14_F_2_N_2_, M.W. 272.30)

Prepared from 4-fluorobenzaldehyde (**1a**) (4.81 mL, 44.87 mmol). Product obtained as a white solid, yield 6.02 g (99%). M.p. 120-122 °C [Lit. M.p. 122-124 °C]^1^, TLC (3:1 petroleum ether/EtOAc), R*f* = 0.84. ^1^H NMR (CDCl_3_): *δ* 8.26 (s, 2H, 2 x CH=N), 7.70 (d, *J* = 7.1 Hz, 4H, Ar), 7.09 (d, *J* = 8.5 Hz, 4H, Ar), 3.97 (s, 4H, 2 x CH_2_).

*N,N´-(Ethane-1,2-diyl)bis(1-(4-chlorophenyl)methanimine) (****3b****)*

(C_16_H_14_Cl_2_N_2_, M.W. 305.20)

Prepared from 4-chlorobenzaldehyde (**1b**) (10.54 mL, 89.75 mmol). Product obtained as a white solid, yield 11.1 g (81%). M.p. 146-148 °C [Lit. M.p. 146-148 °C]^2^, TLC (3:1 petroleum ether/EtOAc), R*f* = 0.68. ^1^H NMR (CDCl_3_): *δ* 8.35 (s, 2H, 2 x CH=N), 7.43 (d, *J* = 8.4 Hz, 4H, Ar), 7.50 (d, *J* = 8.4 Hz, 4H, Ar), 3.88 (s, 4H, 2 x CH_2_).

*N,N´-(Ethane-1,2-diyl)bis(1-(4-bromophenyl)methanimine) (****3c****)*

(C_16_H_14_Br_2_N_2_, M.W. 394.11)

Prepared from 4-bromobenzaldehyde (**1c**) (11.06 g, 59.83 mmol). Product obtained as a white solid, yield 10.6 g (91%). M.p. 140-142 °C [Lit. M.p. 94-96 °C]^1^, TLC (3:1 petroleum ether/EtOAc), R*f* = 0.71. ^1^H NMR (CDCl_3_): *δ* 8.35 (s, 2H, 2 x CH=N), 7.43 (d, *J* = 8.4 Hz, 4H, Ar), 7.50 (d, *J* = 8.4 Hz, 4H, Ar), 3.88 (s, 4H, 2 x CH_2_).

*N,N´-(Ethane-1,2-diyl)bis(1-(4-methoxyphenyl)methanimine) (****3e****)*

(C_18_H_20_N_2_O_2_, M.W. 296.37)

Prepared from 4-anisaldehyde (**1e**) (4.46 mL, 36.72 mmol). Product obtained as a white solid, yield 4.97 g (91%). M.p. 100-102 °C [Lit. M.p. 104 °C]^2^, TLC (2:1 petroleum ether/EtOAc), R*f* = 0.74. ^1^H NMR (CDCl_3_): *δ* 8.23 (s, 2H, 2 x CH=N), 7.66 (d, *J* = 7.7 Hz, 4H, Ar), 6.92 (d, *J* = 7.6 Hz, 4H, Ar), 3.93 (s, 4H, 2 x CH_2_), 3.85 (s, 6H, 2 x OCH_3_).

*N,N´-(Ethane-1,2-diyl)bis(1-(4-isopropylphenyl)methanimine) (****3g****)*

(C_22_H_28_N_2_, M.W. 320.48)

Prepared from cuminaldehyde (**1g**) (10 mL, 65.92 mmol). Product obtained as a yellow solid,^3^ yield 10.52 g (99%). M.p. 56-58 °C, TLC (4:1 petroleum ether/EtOAc), R*f* = 0.91. ^1^H NMR (CDCl_3_): *δ* 8.29 (s, 2H, 2 x CH=N), 7.63 (d, *J* = 8.0 Hz, 4H, Ar), 7.29 (d, *J* = 8.0 Hz, 4H, Ar), 3.84 (s, 4H, 2 x CH_2_), 2.90 (m, 2H, 2 x CH(CH_3_)_2_), 1.19 (d, *J* = 6.9 Hz, 12H, 4 x CH_3_).

*N,N´-(Ethane-1,2-diyl)bis(1-(4-tert-butylphenyl)methanimine) (****3h****)*

(C_24_H_32_N_2_, M.W. 348.53)

Prepared from 4-*tert*-butylbenzaldehyde (**1h**) (7.21 mL, 43.14 mmol). Product obtained as a white solid, yield 7.35 g (98%). M.p. 112-114 °C [Lit. M.p. 115.6-116.8 °C]^4^, TLC (5:1 petroleum ether/EtOAc), R*f* = 0.86. ^1^H NMR (CDCl_3_): *δ* 8.30 (s, 2H, 2 x CH=N), 7.63 (d, *J* = 10.0 Hz, 4H, Ar), 7.44 (d, *J* = 10.0 Hz, 4H, Ar), 3.85 (s, 4H, 2 x CH_2_), 1.28 (s, 18H, 2 x C(CH_3_)_3_).

*N,N´-(Ethane-1,2-diyl)bis(1-(pyridin-4-yl)methanimine) (****3i****)*

(C_14_H_14_N_4_, M.W. 238.29)

Prepared from isonicotinaldehyde (**1i**) (10 mL, 0.106 mol). Product obtained as a yellow solid, yield 11.52 g (91%). M.p. 122-124 °C [Lit. M.p. 123-125 °C]^5^, TLC (2:1 EtOAc/petroleum ether), R*f* = 0.44. ^1^H NMR (DMSO-d_6_): *δ* 8.66 (d, *J* = 3.6 Hz, 4H, pyridine), 8.40 (s, 2H, 2 x CH=N), 7.64 (d, *J* = 3.6 Hz, 4H, pyridine), 3.96 (s, 4H, 2 x CH_2_).

*N,N´-(Ethane-1,2-diyl)bis(1-(pyridin-3-yl)methanimine) (****3j****)*

(C_14_H_14_N_4_, M.W. 238.29)

Prepared from nicotinaldehyde (**1i**) (10 mL, 0.106 mol). Product obtained as a white solid,^6^ yield 11.57 g (91%). M.p. 80-82 °C, TLC (2:1 EtOAc/petroleum ether), R*f* = 0.43. ^1^H NMR (DMSO-d_6_): *δ* 8.85 (s, 2H, pyridine), 8.62 (m, 2H, pyridine), 8.43 (s, 2H, 2 x CH=N), 8.10 (t, *J* = 5.6 Hz, 2H, pyridine), 7.46 (d, *J* = 5.7 Hz, 2H, pyridine), 3.93 (s, 4H, 2 x CH_2_).

***General method for the preparation of diamines (4)****.* To an ice-cooled stirred suspension of diimine (**3**) (1 m.eq.) in methanol (10 mL/mmol) was added sodium borohydride (4.6 m.eq.) in portions. The reaction mixture was stirred for 4 h at room temperature. After the reaction was complete, methanol was evaporated under vacuum and cold water (10 mL/mmol) was added to the remaining residue until cessation of effervescence. The mixture was extracted with EtOAc (10 mL/mmol), and the organic layer was washed with water (2 x 10 mL/mmol), brine (2 x 10 mL/mmol), dried (MgSO_4_) and evaporated under vacuum.

*N^1^,N^2^-Bis(4-fluorobenzyl)ethane-1,2-diamine (****4a****)*

(C_16_H_18_F_2_N_2_, M.W. 276.33)

Prepared from *N,N´*-(ethane-1,2-diyl)bis(1-(4-fluorophenyl)methanimine) (**3a**) (3.25 g, 11.91 mmol). Product obtained as a colourless oil,^1^ yield 2.9 g (88%). TLC (1:1 petroleum ether/EtOAc), R*f* = 0.25. ^1^H NMR (CDCl_3_): *δ* 7.28 (d, *J* = 7.1 Hz, 4H, Ar), 7.01 (d, *J* = 8.0 Hz, 4H, Ar), 3.75 (s, 4H, 2 x CH_2_), 2.75 (s, 4H, 2 x CH_2_), 1.57 (br s, 2H, 2 x NH).

*N^1^,N^2^-Bis(4-chlorobenzyl)ethane-1,2-diamine (****4b****)*

(C_16_H_18_Cl_2_N_2_, M.W. 309.23)

Prepared from *N,N´*-(ethane-1,2-diyl)bis(1-(4-chlorophenyl)methanimine) (**3b**) (6.0 g, 19.65 mmol). Product obtained as a yellow waxy solid,^2^ yield 4.57 g (75%). TLC (1:1 petroleum ether/EtOAc), R*f* = 0.24. ^1^H NMR (CDCl_3_): *δ* 7.33 (m, 8H, Ar), 3.65 (s, 4H, 2 x CH_2_), 2.56 (s, 4H, 2 x CH_2_), 2.05 (br s, 2H, 2 x NH).

*N^1^,N^2^-Bis(4-bromobenzyl)ethane-1,2-diamine (****4c****)*

(C_16_H_18_Br_2_N_2_, M.W. 398.14)

Prepared from *N,N´*-(ethane-1,2-diyl)bis(1-(4-bromophenyl)methanimine) (**3c**) (10.62 g, 29.96 mmol). Product obtained as a pale yellow oil,^1^ yield 9.55 g (89%). TLC (1:1 petroleum ether/EtOAc), R*f* = 0.28. ^1^H NMR (CDCl_3_): *δ* 7.43 (d, *J* = 8.2 Hz, 4H, Ar), 7.19 (d, *J* = 8.2 Hz, 4H, Ar), 3.72 (s, 4H, 2 x CH_2_), 2.72 (s, 4H, 2 x CH_2_), 1.56 (br s, 2H, 2 x NH).

*N^1^,N^2^-Bis(4-methoxybenzyl)ethane-1,2-diamine (****4e****)^2^*

(C_18_H_24_N_2_O_2_, M.W. 300.40)

Prepared from *N,N´*-(ethane-1,2-diyl)bis(1-(4-methoxyphenyl)methanimine) (**3e**) (2.8 g, 9.44 mmol). Product obtained as a yellow waxy solid, yield 2.13 g (75%). TLC (2:1 petroleum ether/EtOAc), R*f* = 0.15. ^1^H NMR (DMSO-d_6_): *δ* 7.22 (d, *J* = 8.1 Hz, 4H, Ar), 6.86 (d, *J* = 8.4, 4H, Ar), 3.73 (s, 6H, 2 x OCH_3_), 3.59 (s, 4H, 2 x CH_2_), 2.55 (s, 4H, 2 x CH_2_), 2.00 (br s, 2H, 2 x NH).

*N^1^,N^2^-Bis(4-ethylbenzyl)ethane-1,2-diamine (****4f****)*

(C_20_H_28_N_2_, M.W. 296.45)

Prepared from *N,N´*-(ethane-1,2-diyl)bis(1-(4-ethylyphenyl)methanimine) (**3f**) (10.5 g, 35.9 mmol). Product obtained as a yellow oil,^7^ yield 10.31 g (97%). TLC (2:1 petroleum ether/EtOAc), R*f* = 0.50. ^1^H NMR (DMSO-d_6_): *δ* 7.20 (d, *J* = 7.9 Hz, 4H, Ar), 7.13 (d, *J* = 7.7 Hz, 4H, Ar), 3.61 (s, 4H, 2 x CH_2_), 2.58 (q, *J* = 7.6 Hz, 4H, 2 x CH_2_CH_3_), 2.51 (s, 4H, 2 x CH_2_), 1.44 (br s, 2H, 2 x NH), 1.16 (t, *J* = 7.6 Hz, 6H, 2 x CH_2_CH_3_).

*N^1^,N^2^-Bis(pyridin-4-yl methyl)ethane-1,2-diamine (****4i****)*

(C_14_H_18_N_4_, M.W. 242.32)

 Prepared from *N,N´*-(ethane-1,2-diyl)bis(1-(pyridine-4-yl)methanimine) (**3i**) (13 g, 54.55 mmol). Product obtained as a yellow oil,^7^ yield 3.85 g (29%). TLC (2:1 petroleum ether/EtOAc), R*f* = 0.12. ^1^H NMR (DMSO-d_6_): *δ* 7.65 (d, *J* = 5.6 Hz, 4H, pyridine), 7.33 (d, *J* = 5.7 Hz, 4H, pyridine), 3.85 (s, 4H, 2 x CH_2_), 3.71 (s, 4H, 2 x CH_2_), 2.46 (br s, 2H, 2 x NH)

**Ethyl 1,4-bis(4-methoxybenzyl)piperazine-2-carboxylate (5e)**

(C_23_H_30_N_2_O_4_, M.W. 398.50)

To a stirred solution of *N*^1^,*N*^2^-bis(4-methoxybenzyl)ethane-1,2-diamine (**4e**) (2.13 g, 7.09 mmol 1 m.eq., 2.84 g, 10.27 mmol) in anhydrous toluene at 80 °C (7.1 mL), was added triethylamine (2.47 mL, 17.73 mmol) and 2,3-dibromopropionic acid ethyl ester (1.08 mL, 7.44 mmol 1.05) dropwise, and the reaction mixture was heated at 80 °C overnight. The solvent was evaporated and the residue extracted with CH_2_Cl_2_ (70 mL), washed with saturated aqueous NaHCO_3_ (5 x 35 mL) and brine (3 x 35 mL). The organic layer was dried (MgSO_4_) and concentrated under reduced pressure and the crude product was purified by gradient flash column chromatography. The product was elutes with petroleum ether – EtOAc 7:3 v/v to give the product as a yellow wax,^8^ yield 2.17 g (78%), TLC (1:1 petroleum ether/EtOAc), R*f* = 0.61. ^1^H NMR (CDCl_3_): *δ* 7.24 (d, *J* = 8.6 Hz, 2H, Ar), 7.20 (d, *J* = 8.5 Hz, 2H, Ar), 6.86 (m, 4H, Ar), 4.16 (q, *J* = 7.1 Hz, 2H, CH_2_CH_3_), 3.87 (d, *J* = 13.0 Hz, 1H, H-7a), 3.78 (s, 6H, 2 x OCH_3_), 3.71 (d, *J* = 13.0 Hz, 1H, H-7_b_), 3.65 (d, *J* = 13.0 Hz, 1H, H-8_a_), 3.54 (d, *J* = 13.0 Hz, 1H, H-8_b_), 3.30 (dd, *J* = 3.5, 6.0 Hz, 1H, H-2), 2.68 (m, 1H, H-3_a_), 2.41 (m, 1H, H-3_b_), 2.30 (m, 4H, H-6_a,b_, H-5_a,b_), 1.25 (t, *J* = 7.1 Hz, 3H, CH_2_CH_3_).

**(1,4-Bis(4-methoxybenzyl)piperazin-2-yl)methanol (6e)**

(C_21_H_28_N_2_O_3_, M.W. 356.47)

To an ice-cooled solution of ethyl 1,4-bis(4-methoxybenzyl)piperazine-2-carboxylate (**5e**) (2.0 g, 5.0 mmol 1 m.eq.) in dry THF (15 mL) was added LiAlH_4_ (1M in THF, 7.5 mL, 7.5 mmol) dropwise over 25 min. The reaction was then stirred at room temperature overnight, then cooled in an ice-bath and carefully quenched with H_2_O until cessation of effervescence. The reaction mixture was extracted with EtOAc (2 x 50 mL), then the combined organic layers washed with H_2_O (3 x 50 mL), dried (MgSO_4_) and concentrated under reduced pressure. The crude product was purified by gradient flash column chromatography and the product was eluted with CH_2_Cl_2_-MeOH 95:5 v/v to give the product as a light brown solid, yield 1.19 g (67%). M.p. 96-98 °C [Lit. M.p. 94-96 °C]^8^, TLC (95:5 CH_2_Cl_2_/MeOH), R*f* = 0.49. ^1^H NMR (DMSO-d_6_): *δ* 7.17 (m, 4H, Ar), 6.86 (m, 4H, Ar), 4.50 (br s, 1H, OH), 3.73 (s, 1H, OCH_3_), 3.72 (s, 1H, OCH_3_), 3.70 (m, 2H, H-9_a,b_), 3.38 (d, *J* = 12.7 Hz, 1H, H-7_a_), 3.33 (d, *J* = 12.7 Hz, 1H, H-7_b_), 3.18 (m, 2H, H-8_a,b_), 2.70 (m, 1H, H-2), 2.56 (m, 1H, H-3_a_), 2.46 (m, 1H, H-3_b_), 2.35 (m, 1H, H-6_a_), 2.05 (m, 3H, H-6_b_, H-5_a,b_).

**2-(Chloromethyl)-1,4-bis(4-methoxybenzyl)piperazine (7e)**

(C_21_H_27_ClN_2_O_2_, M.W. 374.91)

To an ice-cooled solution of (1,4-bis(4-methoxybenzyl)piperazin-2-yl)methanol (**6e**) (0.60 g, 1.60 mmol 1 m.eq.) in dry CH_2_Cl_2_ (8 mL) was added thionyl chloride (1.17 mL, 16 mmol) dropwise over 25 min. The reaction was stirred at room temperature for 48 h and then cooled in an ice-bath and carefully quenched with saturated aqueous NaHCO_3_ in portions until slightly basic (pH 8.0). The organic layer was separated, washed with brine (3 x 16 mL), H_2_O (2 x 16 mL), dried (MgSO_4_) and evaporated under reduced pressure. The crude product was purified by gradient flash column chromatography and the product was eluted with petroleum ether-EtOAc 7:3 v/v to give the product as a white solid, yield 0.45 g (71%). M.p. 82-84 °C [Lit. M.p. 76-82 °C]^8^, TLC (2:1 petroleum ether/EtOAc), R*f* = 0.53. ^1^H NMR (DMSO-d_6_): *δ* 7.20 (m, 4H, Ar), 6.87 (d, *J* = 8.3 Hz, 4H, Ar), 3.93 (m, 2H, H-9_a,b_), 3.83 (m, 2H, H-7_a,b_), 3.73 (s, 6H, 2 x OCH_3_), 3.39 (m, 2H, H-8_a,b_), 2.65 (m, 1H, H-2), 2.58 (m, 1H, H-3_a_), 2.43 (m, 1H, H-3_b_), 2.36 (m, 1H, H-6_a_), 2.20 (m, 3H, H-6_b_, H-5_a,b_).

**References**

(1) Husain, A.; Bhutani, R.; Kumar, D.; Shin, D. Synthesis and biological evaluation

of novel substituted-imidazolidine derivatives. *J. Korean Chem. Soc.* **2013**, *57*, 227

233.

(2) Sharma, V.; Khan, M. S. Y. Synthesis of novel tetrahydroimidazole derivatives and studies for their biological properties. *Eur. J. Med. Chem.* **2001**, *36*, 651-658.

(3) Arish, D.; Nair, M. S. Synthesis, spectroscopic, antimicrobial, DNA binding and cleavage studies of some metal complexes involving symmetrical bidentate N, N donor Schiff base ligand. *Spectrochim. Acta A* **2011**, *82*, 191–199.

(4) Komatsu, H.; Ochiai, B.; Hino, T.; Endo, T. Thermally latent reaction of hemiacetal ester with epoxide controlled by Schiff-base-zinc chloride complexes with tunable catalytic activity. *J. Mol. Catal. A-Chem.* **2007**, *273*, 289–297.

(5) Al-Lami, A. K. Preparation and mesomorphic characterization of supramolecular hydrogen-bonded dimer liquid crystals. *Polycycl. Aromat. Comp.* **2016**, *36*, 197–212.

(6) Ouyang, X-M.; Fei, B-L.; Okamura, T-A.; Bu, H-W.; Sun, W-Y.; Tang, W-X.; Ueyama, N. Syntheses, crystal structures, and properties of four two-dimensional network complexes with multidentate bis(Schiff Base) ligands. *Eur. J. Chem*. **2003**, (4), 618-627.

(7) Buchs, B.; Godin, G.; Trachsel, A.; de Saint Laumer, J-Y.; Lehn, J-M.; Herrmann, A*.* Reversible aminal formation: controlling the evaporation of bioactive volatiles by dynamic combinatorial/covalent chemistry. *Eur. J. Org. Chem.* **2011**, (4) 681–695.

(8) Abd El-wahab, H.A.A.; Accietto, M.; Marino, L.B.; McLean, K.J.; Levy, C.W.; Abdel-Rahman, H.M.; El-Gendy, M.A.; Munro, A.W.; Aboraia, A.S.; Simons, C. Design, synthesis and evaluation against *Mycobacterium tuberculosis* of azole piperazine derivatives as dicyclotyrosine (cYY) mimics. *Bioorg. Med. Chem*. **2018**, *26*, 161-176.


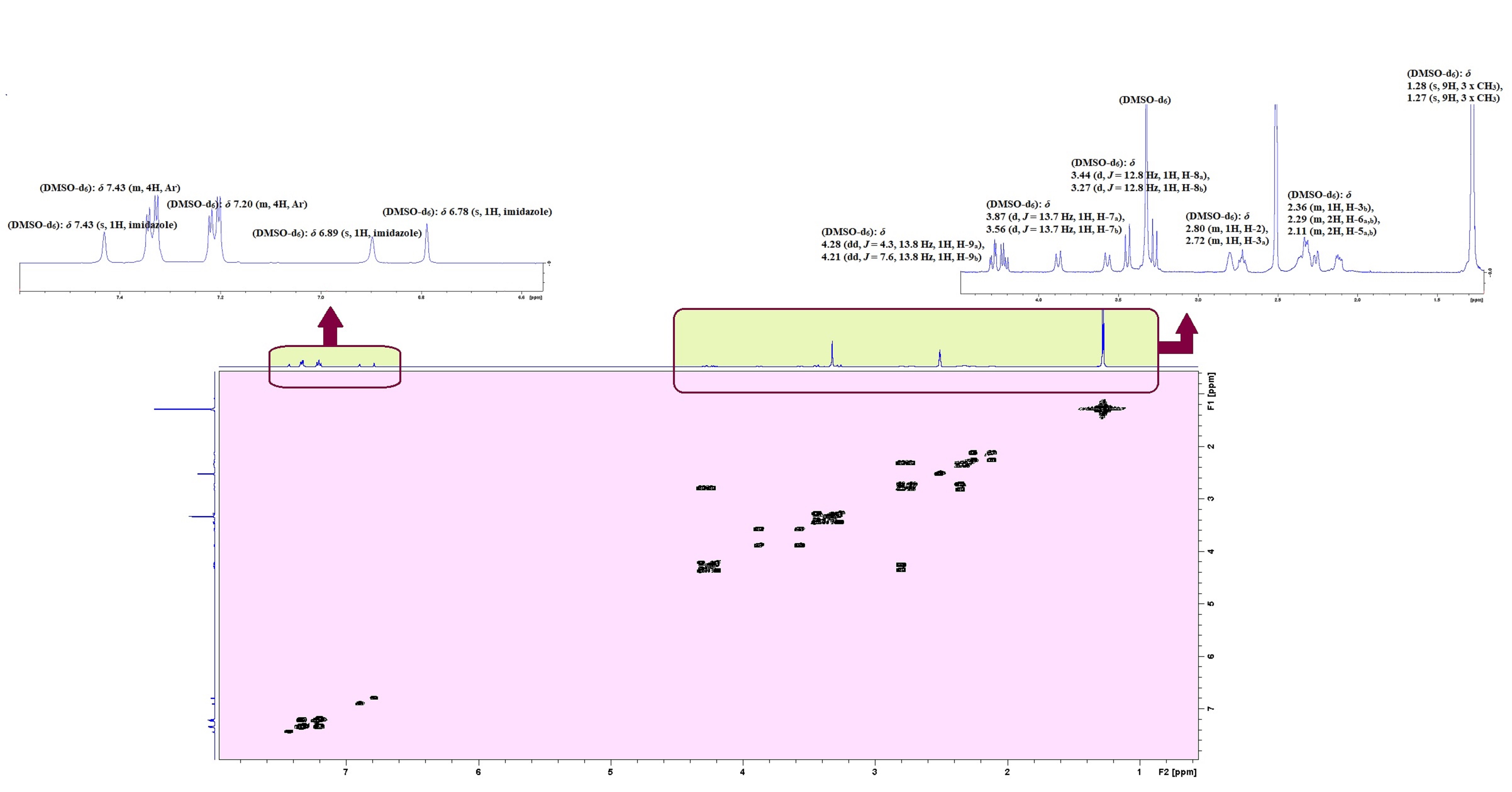


**Figure S1**. 2-((1*H*-Imidazol-1-yl)methyl)-1,4-bis(4-(tert-butyl)benzyl)piperazine (**8h**) 1D (^1^H) and 2D (COSY) NMR spectra illustrating the coupling interactions between protons
